# Modeling and computational study of cancer treatment with radiotherapy using real data

**DOI:** 10.1371/journal.pone.0320906

**Published:** 2025-05-20

**Authors:** Parvaiz Ahmad Naik, Muhammad Farman, Saba Jamil, Muhammad Umer Saleem, Kottakkaran Sooppy Nisar, Zhengxin Huang

**Affiliations:** 1 Department of Mathematics and Computer Science, Youjiang Medical University for Nationalities, Baise, Guangxi, China; 2 Art and Science Faculty, Department of Mathematics, Near East University, Boulevard, Nicosia/Mersin, Turkey; 3 Research Center of Applied Mathematics, Khazar University, Baku, Azerbaijan; 4 Institute of Mathematics, Khwaja Fareed University of Engineering and Information Technology, Rahim Yar Khan, Pakistan; 5 Department of Mathematics, University of Education, Lahore, Pakistan; 6 Department of Mathematics, College of Science and Humanities, Prince Sattam bin Abdulaziz University, Al Kharj, Saudi Arabia; Kwame Nkrumah University of Science and Technology, GHANA

## Abstract

The numerical simulation of biological processes with non-integer ordering is attracting an increasing amount of interest from scientists and academics. Traditional biological systems can be presented in a fixed order, but fractional-order derivative systems are not considered stable orders. When the fractional derivative has a non-fixed order, it becomes more useful for simulating real-world problems. In this paper, we aim to study the dynamics of a novel technique that we propose, implement, and use in a radiation model for the treatment of cancer. We present some intriguing results for the cancer treatment fractal fractional model in the context of this innovative operator. Research has been done on the cancer model in both qualitative and quantitative manners. The first and second derivatives of the Lyapunov function are used to analyze the stability of the cancer fractal fractional model. Using the linear growth theory, the existence of a unique solution has been derived under the FFM. Lagrangian-piece-wise interpolation has been used to obtain numerical results for various fractal-fractional operators. The fractal fractional model was used to simulate the treatment process of three patients. Different values of fractional order μ, fractal dimension ν, and other parameter values have been used to show the graphs. Additionally, we looked at how radiation changed both healthy cells and malignant cells over time. The study confirmed the effectiveness of radiation medicine against populations as well as the occurrence of the memory effect during μ and ν transitions from 1. A biological process requires fractal-fractional processes which provide superior modeling capabilities compared to traditional fractional operators as well as classical operators. This research brings novel significance through its implementation of fractal-fractional operators as they provide a superior approach to model cancer treatment processes by better representing biological system complexities. Standard modeling systems cannot reproduce both important memory dynamics together with non-local communication patterns which play essential roles in cancer development and treatment analysis. The implementation of fractal-fractional derivatives enables our model to produce a more realistic representation of cancer cell and healthy cell radiotherapy responses throughout time. Our study has upgraded theoretical cancer dynamic analysis and developed optimized treatment methods for customization purposes. Wider understanding of cancer cell reactions to treatments enables healthcare providers to adopt personalized strategies that produce superior recovery outcomes for their patients. The model acquires stability strength through Lyapunov functions analysis to create a solid scientific foundation in oncology research.

## 1 Introduction

Cancer remains among the leading causes of death worldwide since it affects human populations significantly, affecting public health. The second highest cause of death exists globally behind cardiovascular diseases as per data in [1]. The cancer development risk grows as people age because most cancer diagnoses happen to individuals who surpass 55 years of age according to research in [2]. Research data indicates that the lifetime chances of getting cancer amount to 50% for males and 33% for females across numerous geographical areas [3]. Researcher communities from various fields have been motivated to develop successful cancer treatments because of these alarming statistics. The development of advanced cancer therapy requires knowledge about the cell characteristics of malignant cells since they determine the disease’s aggressive behavior. Radiotherapy emerged as a leading economical therapeutic method among cancer treatment approaches since it consumes less than 5% of the total medical expenses [4].

To simulate the effects of radiotherapy on tumor progression, mathematical models have been developed [5–7]. A variety of mathematical models have also been suggested to simulate chemotherapy treatment [8–11]. Numerous research [12–14] have also offered mathematical models for the radiotherapy and chemotherapy used to treat cancer.

In various crucial disciplines, including physics, economics, engineering, biology, medicine, and many others, fractional calculus depicts dynamic processes [15–21]. The list of fractional derivatives can be split into two categories. The fractional derivatives of the SK (Singular Kernel) are those of Katugampola, Riemann-Liouville, and Caputo. The fractional derivative without SK is Caputo-Fabrizio and the ABC operator. Differential equations and fractional order integrals have an incredible impact on modeling and simulation using these three different types of kernels [22–24]. Most of these problems lack an exact analytical solution. Many scholars are naturally drawn to investigate this scenario and use numerical and approximative methods to find solutions. Numerous helpful techniques exist, including homotopy analysis [25–27], Laguerre polynomial [28], a modified predictor-corrector method [29], natural decomposition method [30], lagrangian interpolation [31], and others.

Recent work by Atangana [31]et al. provided a fractal-fractional derivative of the convolution of the power law, the exponential decay law, and the generalized Mittag-Leffler function, which are three notions that are already known. In addition to considering fractal impact, memory, and non-locality, they combine the concepts of fractal derivative and fractional differentiation. The power law ignored memory, and self-similar hybrid behavior problems can all be made clear using the fractal-fractional operators. However, it has been demonstrated that said operator can better represent the complex behavior of various real-world phenomena [33–36]. Although these fractional derivatives show many benefits, they are not appropriate in all circumstances. A constant’s Riemann-Liouville derivative is not zero. Any function with zero at the origin, such as the exponential and Mittag-Leffler functions, has a singularity in its fractional derivation. These disadvantages reduce the field of application of the fractional derivative. Compared to integer order differential equations, the mathematical models using fractional differential equations are highly helpful in explaining the development of tumors and the interactions between tumors and host cells. Numerous articles about the mathematical modeling of cancer tumors have been written recently. The elements of immunity are crucial in the therapy of cancer and have been described by numerous mathematical models [38] provided by Dokuyucu *et al*. [37] and are known as the fractional radiation cancer treatment model. The Caputo-Fabrizio fractional derivative was incorporated into the preexisting cancer therapy model by Dokuyucu *et al*. [37]. The authors established the requirements for the existence and uniqueness of the model’s solutions using the fixed-point theory. The authors demonstrated that the proposed fractional cancer therapy model has a singularly positive solution based on the stated criteria. The same concept was given similarly by Awadalla *et al*. [39]. However, they used a different kind of fractional derivative. The Hadamard fractional derivative was incorporated into the cancer therapy model [39, 40].

The qualitative examination of non-linear systems exposes the possibility of situations that could result in physical, chemical, or biological phenomena. A well-known issue in the theory of integer ordinary differential equations, the analysis of stability or instability of the equilibrium points, arises in classifying these scenarios. On the one hand, the Lyapunov indirect technique, also known as the linearization method, uses the concept of system linearization around a certain equilibrium point, and one may ascertain the local behavior of the point. On the other hand, the Lyapunov direct technique is the most crucial tool for nonlinear system analysis since it allows one to ascertain the overall behavior of the equilibrium point without having to develop system solutions. The method uses Lyapunov functions as additional operational tools. Stability features can always be established through suitable Lyapunov functions according to classical stability theorems in this method. The asymptotic stability analysis uses classical Lyapunov theorem and LaSalle’s invariance principle while instability analysis relies mainly on classical Chetaev theorem [41].

The authors presented a fractional-order vaccination model for Tuberculosis [42] to evaluate how vaccination measures affect disease transmission in people with medical conditions. The research findings demonstrate how fractional-order dynamics produce more precise disease prediction outcomes and confirm the necessity of specific vaccination plans to lower TB prevalence. Using double-dose vaccination as a prevention has shown a remarkable decrease in measles transmission according to research [43]. The particular mathematical model utilizing fractional derivatives yielded enhanced understanding of disease propagation patterns and vaccination program outcomes. By using Fractal-Fractional order derivatives researchers successfully model age-structured smoking dynamics and establish how government intervention specifically targets smoking behavior for various age ranges [44]. Research in [45] shows the Atangana-Baleanu Caputo fractional-order operator gives better accuracy to diabetes mellitus dynamics to examine disease evolution and therapeutic approaches. Fractional-order modeling produces accurate results about co-infection of malaria and COVID-19, which illustrates simultaneous transmission effects and recommends combined disease control methods [46].

A global dynamics approach combined with computational modeling represents an important key for researching alcohol addiction because of its vital public health outcomes [47]. The study establishes an advanced computational system to examine emerging zoonotic viral infection patterns and control measures by adopting creative epidemic modeling techniques [48]. Using fractional and fractal-fractional operators with a power-law kernel, researchers study human-to-animal transmission dynamics of monkeypox infection in zoonotic disease settings [49]. The field of mathematical epidemiology experienced major growth through fractal-fractional approach-based modeling of coronavirus and HIV/AIDS dynamics per a study published in 2022 [50, 51].

The research develops a fractal fractional-order mathematical model of radiotherapy-based cancer treatment derived from the previously discussed findings. Multiple outcomes resulted from running treatments on cancer patients while changing starting values and parametric orders and dimensional parameters. The study utilized parameter and starting value information from [7, 40] to obtain solutions for treating three individual cancer patients. Researchers proceed to change the initial values for each patient before recording the updated solutions. Fractal-fractional modeling prevails as a superior replacement for traditional fractional models since it optimally characterizes biological system dynamics within cancer treatment scenarios. The Markovian assumptions in classical models exclude long-term dependencies whereas fractional models include memory features without adequate spatial irregularity adjustments. A fractal-fractional operator combines fractal properties and memory features, which creates an improved representation framework for tumor development alongside radiotherapy reactions. Fractal components in mathematical models let researchers represent the primitive and nonuniform arrangement of tumor cells because classical derivatives work only for smooth geometric structures. The numerical stability of Atangana-Baleanu operator becomes more accurate because of its Mittag-Leffler kernel which performs better than singular kernels found in conventional fractional derivatives. This model demonstrates practical significance for clinical use because its adjustable fractional order parameters and fractal dimensions enable customized predictions during treatment. This framework produces effective descriptions of both healthy cell and malignant cell nonlocal interactions in treatments involving radiotherapy. The adaptable fractal-fractional framework leads to better numerical stability and convergence, which produces more dependable simulations. This method surpasses existing models because it includes fractal geometry together with memory effects as a means to accurately model cancer progression complexity. As a result, it delivers a versatile solution to maximize radiotherapy plans and produce superior clinical results for mathematical oncology.

By employing fractional differential operators, cancer treatment through radiotherapy becomes enhanced through more accurate representations of complex biological processes, including anomalous diffusion of nutrients and drugs in tumor tissues. Fractional differential operators enhance cancer treatment with radiotherapy because they capture the combined effects of memory mechanisms and long-distance interactions. Fractional modeling produces better disease prediction models, which results in optimized treatment plans based on radiation dosing schedules. Through their use fractional models enable better treatment results together with reduced harm to healthy tissue structures. The singular kernels used by fractional operators differ from standard integer derivatives because they retain memory attributes which enable accurate modeling of disease transmission through past state dependencies and long-range relationships. Such operators are more efficient at modeling biological systems that exhibit non-local interactions and complex anomalous diffusion processes through their ability to include memory effects. The usage of fractional operators with singular kernels like Caputo derivatives brings memory effects to infected compartments thus modifying their stability dynamics by adding non-local interactions and affecting their equilibrium convergence rates. When fractional order values are lower in the system, the convergence rate becomes slower, thus infected compartments exhibit longer duration compared to traditional models. This study is important as it introduces a novel fractal-fractional model for cancer radiotherapy, offering a more accurate representation of biological processes with memory effects. The key results include the demonstration of the model’s stability and effectiveness in simulating cancer treatment dynamics, showing that radiation dosage impacts cancer and healthy cells differently. The conclusions highlight that the fractal-fractional approach provides deeper insights into cancer dynamics, making it a valuable tool for modeling and optimizing radiotherapy treatments. This model outperforms classical and fractional-order models in capturing complex real-world phenomena.

The paper’s contents are organized as follows: The fundamental ideas of fractal-fractional calculus are covered in Sect 2. Sect 3 investigates the model solutions’ positive invariance and boundedness. The 4 sections analyze the local and global asymptotic stability of the fractal fractional order cancer treatment model. We use the first and second derivatives of the Lyapunov function to analyze the global asymptotic stability of the fractal fractional order cancer treatment model. The uniqueness and existence theory analysis of the suggested model is covered in Sect 5. The numerical results we obtained using the Lagrangian-piece-wise interpolation approach are presented in Sect 6. The numerical simulations of the theoretical results and a discussion of the graphs are presented in Sect 7. The article’s conclusion is found in Sect 8.

## 2 Basic concepts

This section consists of some important definitions.

**Definition 2.1** [31, 32]

The FFP derivative of *z*(*t*) is defined as follows:

$${\;}^{FFP}D_{0,t}^{\alpha ,\alpha_{1}}(z(t))=\frac{1}{\Gamma (1-\alpha )}\frac{d}{dt^{\alpha_{1}}}\int_{0}^{t}{\left(t-\xi \right)}^{-\alpha }z(\xi )d\xi ,\;0<\alpha \leq 1,\;0<\alpha_{1}\leq 1,$$ (1)

where

$$\frac{d}{d\xi^{\alpha_{1}}}z(\xi )=\lim_{t\to \xi }\frac{z(t)-z(\xi )}{t^{2-\alpha_{1}}-\xi^{2-\alpha_{1}}}\times (2-\alpha_{1}).$$ (2)

**Definition 2.2** [31, 32]

Following is the definition of the related FF integral operator with power law kernel:

$${\;}^{FFP}I_{0,t}^{\alpha ,\alpha_{1}}(z(t))=\frac{1}{\Gamma (\alpha )}\int_{0}^{t}{\left(t-\xi \right)}^{\alpha -1}\xi^{\alpha_{1}-1}z(\xi )d\xi ,\;0<\alpha \leq 1,\;0<\alpha_{1}\leq 1.$$ (3)

**Definition 2.3** [31, 32]

The FFE derivative of *z*(*t*) is defined as:

$${\;}^{FFE}D_{0,t}^{\alpha ,\alpha_{1}}(z(t))=\frac{M(\alpha )}{1-\alpha }\frac{d}{dt^{\alpha_{1}}}\int_{0}^{t}exp\left[-\frac{\alpha }{1-\alpha }\left(t-\xi \right)\right]z(\xi )d\xi ,\;0<\alpha \leq 1,\;0<\alpha_{1}\leq 1.$$ (4)

**Definition 2.4** [31]

The following is a description of the related FF integral operator with exponential kernel:

$${\;}^{FFE}I_{0,t}^{\alpha ,\alpha_{1}}(z(t))=\frac{\left(1-\alpha )t^{1-\alpha_{1}}z(t\right)}{M(\alpha )}+\frac{\alpha }{M(\alpha )}\int_{0}^{t}\xi^{1-\alpha_{1}}z(\xi )d\xi ,\;0<\alpha \leq 1,\;0<\alpha_{1}\leq 1.$$ (5)

**Definition 2.5** [31]

The FFM derivative of z(t) is defined as follows:

$${\;}^{FFM}D_{0,t}^{\alpha ,\alpha_{1}}(z(t))=\frac{AB(\alpha )}{1-\alpha }\frac{d}{dt^{\alpha_{1}}}\int_{0}^{t}E_{\alpha }\left[-\frac{\alpha }{1-\alpha }{\left(t-\xi \right)}^{\alpha }\right]z(\xi )d\xi ,$$ (6)

where $0<\alpha ,\alpha_{1}\leq 1$ and $AB(\alpha )=1-\alpha +\frac{\alpha }{\Gamma (\alpha )}.$

**Definition 2.6** [31]

The related FF integral operator with Mittag-Lefrel kernel is given by:

$${\;}^{FFM}I_{0,t}^{\alpha ,\alpha_{1}}(z(t))=\frac{\left(1-\alpha )t^{1-\alpha_{1}}z(t\right)}{AB(\alpha )}+\frac{\alpha }{AB(\alpha )\Gamma (\alpha )}\int_{0}^{t}\xi^{1-\alpha_{1}}{\left(t-\xi \right)}^{\alpha -1}z(\xi )d\xi .$$ (7)

## 3 Fractal fractional order cancer model

In this section, we propose a memory-affected fractal fractional-order model for cancer. The cancer epidemic model presented in [7] is a classical derivative to be considered. The radiation strategy is γ, *x*_1_ representing the proportion of healthy cells and *x*_2_ representing the proportion of malignant cells. According to our presumption, γ>0 denotes a treatment stage, whereas γ=0 denotes no treatment stage. The healthy cells are also harmed during the radiation treatment for cancer. The radiation percentage is εγ, ε>0 (ε=0 is the ideal, but it’s not attainable in practice). The related proliferation coefficients, carrying capacities, and competition coefficients are denoted by the terms $\alpha_{i}>0\;(i=1,2)$, $K_{i}>0\;(i=1,2)$, and $\beta_{i}>0\;(i=1,2)$ respectively. The study adopts the classical case model introduced in [7] who developed a radiotherapy-based cancer treatment model through control theory. The research develops the popular model from previous studies by integrating fractal and fractional framework elements for enhanced cancer progression and treatment representations. This modification introduces fractal-fractional derivatives which add both memory effects and fractal characteristics into the model. A significant breakthrough emerges from this study because it developed a fractal-fractional extension that builds upon the classical model. The implementation of the Atangana-Baleanu fractal-fractional derivative enhances biological process modeling accuracy when examining cancer treatment, especially radiotherapy applications.

Biological evidence supports the assumption that healthy cells receive radiation damage since this leads to side effects in normal tissues during treatment. The battle between cancer cells and healthy cells matches what occurs in tumors which normally constrain the growth of normal tissue. Fractional differential operators improve the accuracy of the model because they depict the ongoing interactions as well as the history-based dynamics of cancer growth and radiation responsiveness. The approach leads to improved predictions regarding tumor progression together with clinical reaction outcomes throughout the treatment course. Fractional differential models supply a greater level of accuracy when simulating cancer treatment dynamics when contrasted with traditional integer-order models. The model surpasses current models using fractal-fractional derivatives that reflect biological processes better by capturing non-local dynamics and memory effects. Healthcare professionals can utilize the model for more accurate cancer treatment outcome simulations because of its ability to surpass classical and fractional-order operational methods. A fractal-fractional order model becomes our proposal for measuring intricate interactions between healthy cells and malignant cells under radiotherapy while including both spatial heterogeneity and memory effects. The model builds upon basic and fractional-order cancer models through a fractal-fractional operator that enhances biological sophistication in modeling representation. The system is governed by the following biological parameters:

- $\alpha_{1}$: Growth rate of healthy cells.
- $\alpha_{2}$: Growth rate of malignant cells.
- *K*_1_: Carrying capacity of healthy cells, representing the maximum sustainable population.
- *K*_2_: Carrying capacity of malignant cells, defining their upper population limit.
- $\beta_{1}$: Competition coefficient of malignant cells affecting healthy cells.
- $\beta_{2}$: Competition coefficient of healthy cells affecting malignant cells.
- γ: Radiation treatment intensity, affecting the decline rate of cancer cells.
- ε: Fraction of healthy cells affected by radiation therapy (collateral damage).
- μ: Fractional order parameter, controlling the memory effect of the system.
- ν: Fractal dimension, representing the irregularity of biological tissue structure.

The conversion of the classical cancer treatment model into the fractal-fractional framework involves several systematic steps. Initially, the classical model, based on ordinary differential equations (ODEs) describing the dynamics of healthy and cancer cells under radiotherapy, is established. To adapt this model, the classical time derivative is replaced by a fractal-fractional derivative, which integrates fractional derivatives to capture memory effects and fractal dimensions to reflect self-similarity and complex biological processes. The fractal-fractional derivative is defined using the Mittag-Leffler function and a normalization factor, and it incorporates both fractional order and fractal dimension. The system equations are then rewritten with this new derivative, transforming the model into a fractal-fractional form.

**With Treatment:**

$$\begin{array}{c} {\;}^{FFM}D_{0,t}^{\mu ,\nu }(x_{1}(t))=\alpha_{1}x_{1}\left(1-\frac{x_{1}}{K_{1}}\right)-\beta_{1}x_{1}x_{2}-\varepsilon \gamma x_{1},{\;}^{FFM}D_{0,t}^{\mu ,\nu }(x_{2}(t))=\alpha_{2}x_{2}\left(1-\frac{x_{2}}{K_{2}}\right)-\beta_{2}x_{1}x_{2}-\gamma x_{2}. \end{array}$$ (8)

**Without Treatment (γ=0):**

$$\begin{array}{c} {\;}^{FFM}D_{0,t}^{\mu ,\nu }(x_{1}(t))=\alpha_{1}x_{1}\left(1-\frac{x_{1}}{K_{1}}\right)-\beta_{1}x_{1}x_{2},{\;}^{FFM}D_{0,t}^{\mu ,\nu }(x_{2}(t))=\alpha_{2}x_{2}\left(1-\frac{x_{2}}{K_{2}}\right)-\beta_{2}x_{1}x_{2}, \end{array}$$ (9)

with initial conditions: $x_{1}(0)\geq 0\;,\;\;\;x_{2}(0)\geq 0$

### 3.1 Equilibrium points

For the model (8), the following are the equilibrium points

$$E^{0}=\left(x_{1}^{0},\;x_{2}^{0}\right)=\left(\frac{K_{1}\alpha_{1}-K_{1}\gamma \varepsilon }{\alpha_{1}},\;0\right),$$ (10)

and

$$E^{*}=\left(x_{1}^{*},\;x_{2}^{*}\right)=\left(0,\;\frac{K_{1}\alpha_{2}-K_{1}\gamma }{\alpha_{2}}\right).$$ (11)

### 3.2 Boundedness and positive invariance

From the model (8), we get

$${\dot{x}}_{1}=\alpha_{1}x_{1}\left(1-\frac{x_{1}}{K_{1}}\right)-\beta_{1}x_{1}x_{2}-\varepsilon \gamma x_{1}\leq \alpha_{1}x_{1}\left(1-\frac{x_{1}}{K_{1}}\right),$$ (12)

integration of the above leads to

$$x_{1}(t)\leq \frac{1}{\frac{1}{K_{1}}+x_{1}(0)e^{-\alpha_{1}t}}\Rightarrow \lim_{t\to \infty }\sup(x_{1}(t))\leq K_{1},$$ (13)

Furthermore,

$${\dot{x}}_{2}=\alpha_{2}x_{2}\left(1-\frac{x_{2}}{K_{2}}\right)-\beta_{2}x_{1}x_{2}-\gamma x_{2},$$ (14)

Proceeding as above, we have

$$x_{2}(t)\leq \frac{1}{\frac{1}{K_{2}}+x_{2}(0)e^{-\alpha_{2}t}}\Rightarrow \lim_{t\to \infty }\sup(x_{2}(t))\leq K_{2},$$ (15)

with the initial conditions *x*_1_(0)>0 and *x*_2_(0)>0.

$$\Delta =\left\{\left(x_{1}(t),x_{2}(t)\in R_{+}^{2}\right):0\leq x_{1}(t)\leq K_{1},0\leq x_{2}(t)\leq K_{2}\right\}.$$ (16)

So, the solutions of system (8) are bounded.

### 3.3 Threshold parameters

The Basic Reproduction Number (*R*_0_) is a key indicator for evaluating the dynamics of malignant cell growth and the efficacy of cancer therapies such as radiotherapy. By incorporating this concept into mathematical models, researchers and clinicians gain valuable insights into tumor behavior, enabling them to refine treatment strategies for better patient outcomes. Manipulating factors that influence *R*_0_ through targeted therapeutic interventions can improve the management of cancer as a chronic disease. To better understand the stability conditions of the system, we define the following threshold parameters:

- **Basic Reproduction Ratio of Malignant Cells (*R***_**0**_):
  $$R_{0}=\frac{\alpha_{2}}{\gamma +\beta_{2}K_{1}}$$ (17)
  which determines whether cancer cells will persist or be eradicated under treatment.
- **Net Growth Rate of Healthy Cells (*R***_***h***_):
  $$R_{h}=\frac{\alpha_{1}-\varepsilon \gamma }{\beta_{1}K_{2}}$$ (18)
  which assesses the ability of healthy cells to outcompete malignant cells.

**Remarks 1.**

1. Radiation Effectiveness Threshold (γϵ): Determines the balance between radiation effectiveness and collateral damage to healthy cells. Excessive values may destabilize the system.
2. Healthy Cell Growth Rate ($\alpha_{1}$): Defines the natural proliferation rate of healthy cells, serving as a benchmark for assessing radiation effects.
3. Cancer Cell Growth Rate ($\alpha_{2}$): Represents the intrinsic growth rate of cancer cells, influencing their ability to outcompete healthy cells.
4. Competition Coefficients ($\beta_{1},\beta_{2}$): Measure intercellular competition, where higher values indicate stronger inhibitory effects between healthy and cancer cells.
5. Carrying Capacities ($K_{1},K_{2}$): Establish the maximum sustainable populations for healthy and cancer cells, setting ecological limits in the system.

## 4 Stability

We are interested in understanding the nature of the stability of the equilibrium points of the proposed model in this section.

**Theorem 4.1.** Let $\lambda_{i},i=1,2.$, represent the roots of the characteristic polynomial equation (eigenvalues) connected to the Jacobian matrix of the model (8) at the equilibrium point under investigation. If


$$\left|\arg\lambda_{i}\right|>\mu \frac{\pi }{2},$$

local stability exists at the equilibrium point [52]. At *E*_0_, the Jacobian matrix of (8) is as follows:


$$J(E_{0})=\left[\begin{array}{cc} \gamma \varepsilon -\alpha_{1} & \frac{-\beta_{1}K_{1}(\alpha_{1}-\gamma \varepsilon )}{\alpha_{1}}\\ 0 & \frac{-\beta_{1}K_{1}(\alpha_{1}-\gamma \varepsilon )}{\alpha_{1}}-\gamma \end{array}\right],$$

and, consequently, the eigenvalues are


$$\lambda_{1}=\gamma \varepsilon -\alpha_{1},\lambda_{2}=-\gamma -\beta_{1}K_{1}(\alpha_{1}-\gamma \varepsilon ).$$

**Theorem 4.2.** The equilibrium point *E*_0_ of the model (8) is locally stable if, and only if, $\gamma \varepsilon <\alpha_{1}$.

**Remark 2.** The conditions established in these theorems are essential for understanding how tumors respond to treatment over time. By analyzing local stability, researchers can predict whether a tumor will continue to grow or stabilize under specific treatment protocols. If the basic reproduction number (*R*_0_) related to malignant cells is greater than one, it signifies that malignant cells can proliferate uncontrollably, resulting in instability. On the other hand, if the conditions outlined in Theorem 4.2 are satisfied, it suggests that the treatment can effectively control tumor growth, leading to a more stable tumor environment.

### 4.1 Lyapunov function for global stability

The Lyapunov function is given as follows:

$$L(x_{1}^{*},x_{2}^{*})=\left(x_{1}-x_{1}^{*}-x_{1}^{*}\ln\frac{x_{1}}{x_{1}^{*}}\right)+\left(x_{2}-x_{2}^{*}-x_{2}^{*}\ln\frac{x_{2}}{x_{2}^{*}}\right)$$ (19)

Differentiate both sides with respect to *t*, we obtain

$$\frac{dL}{dt}=\dot{L}=(\frac{x_{1}-x_{1}^{*}}{x_{1}}){\dot{x}}_{1}+(\frac{x_{2}-x_{2}^{*}}{x_{2}}){\dot{x}}_{2}.$$ (20)

Now, we can write their values for derivatives as follows

$$\begin{array}{c} \frac{dL}{dt}=\dot{L}=(\frac{x_{1}-x_{1}^{*}}{x_{1}})\left[\alpha_{1}x_{1}\left(1-\frac{x_{1}}{K_{1}}\right)-\beta_{1}x_{1}x_{2}-\varepsilon \gamma x_{1}\right]\\ \;+(\frac{x_{2}-x_{2}^{*}}{x_{2}})\left[\alpha_{2}x_{2}\left(1-\frac{x_{2}}{K_{2}}\right)-\beta_{2}x_{1}x_{2}-\gamma x_{2}\right]. \end{array}$$ (21)

Substituting $x_{1}=x_{1}-x_{1}^{*}$, $x_{2}=x_{2}-x_{2}^{*}$, leads to:

$$\begin{array}{ccc} \frac{dL}{dt} & = & \dot{L}=\left(\frac{x_{1}-x_{1}^{*}}{x_{1}}\right)\left[\alpha_{1}(x_{1}-x_{1}^{*})\left(1-\frac{\left(x_{1}-x_{1}^{*}\right)}{K_{1}}\right)-\beta_{1}(x_{1}-x_{1}^{*})\left(x_{2}-x_{2}^{*}\right)-\varepsilon \gamma (x_{1}-x_{1}^{*})\right]\\ & & +\left(\frac{x_{2}-x_{2}^{*}}{x_{2}}\right)\left[\alpha_{2}(x_{2}-x_{2}^{*})\left(1-\frac{\left(x_{2}-x_{2}^{*}\right)}{K_{2}}\right)-\beta_{2}(x_{1}-x_{1}^{*})(x_{2}-x_{2}^{*})-\gamma (x_{2}-x_{2}^{*})\right]. \end{array}$$ (22)

$$\begin{array}{ccc} \frac{dL}{dt} & = & \dot{L}=\frac{{\left(x_{1}-x_{1}^{*}\right)}^{2}\alpha_{1}}{x_{1}}-\frac{{\left(x_{1}-x_{1}^{*}\right)}^{2}\alpha_{1}}{x_{1}K_{1}}-\frac{{\left(x_{1}-x_{1}^{*}\right)}^{2}x_{2}\beta_{1}}{x_{1}}+\frac{{\left(x_{1}-x_{1}^{*}\right)}^{2}x_{2}^{*}\beta_{1}}{x_{1}}-\frac{{\left(x_{1}-x_{1}^{*}\right)}^{2}\varepsilon \gamma }{x_{1}}\\ & & +\frac{{\left(x_{2}-x_{2}^{*}\right)}^{2}\alpha_{2}}{x_{2}}-\frac{{\left(x_{2}-x_{2}^{*}\right)}^{2}\alpha_{2}}{x_{2}K_{2}}-\frac{{\left(x_{2}-x_{2}^{*}\right)}^{2}x_{1}\beta_{2}}{x_{2}}+\frac{{\left(x_{2}-x_{2}^{*}\right)}^{2}x_{1}^{*}\beta_{2}}{x_{2}}-\frac{{\left(x_{2}-x_{2}^{*}\right)}^{2}\gamma }{x_{2}}. \end{array}$$ (23)

This can be written as

$$\frac{dL}{dt}=\Upsilon_{1}-\Upsilon_{2},$$ (24)

where

$$\Upsilon_{1}=\frac{{\left(x_{1}-x_{1}^{*}\right)}^{2}\alpha_{1}}{x_{1}}+\frac{{\left(x_{1}-x_{1}^{*}\right)}^{2}x_{2}^{*}\beta_{1}}{x_{1}}+\frac{{\left(x_{2}-x_{2}^{*}\right)}^{2}\alpha_{2}}{x_{2}}+\frac{{\left(x_{2}-x_{2}^{*}\right)}^{2}x_{1}^{*}\beta_{2}}{x_{2}},$$ (25)

and

$$\begin{array}{ccc} \Upsilon_{2} & = & \frac{{\left(x_{1}-x_{1}^{*}\right)}^{2}\alpha_{1}}{x_{1}K_{1}}+\frac{{\left(x_{1}-x_{1}^{*}\right)}^{2}x_{2}\beta_{1}}{x_{1}}+\frac{{\left(x_{1}-x_{1}^{*}\right)}^{2}\varepsilon \gamma }{x_{1}}\\ & & +\frac{{\left(x_{2}-x_{2}^{*}\right)}^{2}\alpha_{2}}{x_{2}K_{2}}+\frac{{\left(x_{2}-x_{2}^{*}\right)}^{2}x_{1}\beta_{2}}{x_{2}}+\frac{{\left(x_{2}-x_{2}^{*}\right)}^{2}\gamma }{x_{2}}. \end{array}$$ (26)

$$0=\Upsilon_{1}-\Upsilon_{2}\Rightarrow \frac{dL}{dt}=0$$ (27)

The greatest compact invariant set for the proposed model is, as can be seen,

$$\left\{\left(x_{1}^{*},x_{2}^{*}\right)\in \Omega :\frac{dL}{dt}=0\right\}$$ (28)

$\left\{E^{*}\right\}$ represents the equilibrium of the nonlinear model. The Lasalle’s invariance principle demonstrates that E* is globally asymptotic and stable in Ω if $\Upsilon_{1}<\Upsilon_{2}$.

**Remarks 3.** The condition $\Upsilon_{1}<\Upsilon_{2}$ in Eq (24) ensures the global asymptotic stability of the fractal-fractional cancer treatment model by using the Lyapunov function. Mathematically, the Lyapunov function $L(x_{1},x_{2})$ is designed to be positive definite, meaning $L(x_{1},x_{2})>0$ for $\left(x_{1},x_{2})\neq (x_{1}^{*},x_{2}^{*}\right)$ and $L(x_{1}^{*},x_{2}^{*})=0$ at the equilibrium point. The time derivative of $L(x_{1},x_{2})$, $\frac{dL}{dt}$, measures how the "energy" of the system changes. Decomposing $\frac{dL}{dt}$ into positive ($\Upsilon_{1}$) and negative ($\Upsilon_{2}$) terms, we require $\Upsilon_{1}<\Upsilon_{2}$ for global stability. This ensures that the stabilizing factors dominate, making $\frac{dL}{dt}<0$ and driving the system toward the equilibrium point. This condition is mathematically justified through the positive definiteness of $L(x_{1},x_{2})$ and its derivative, and is verified by numerical simulations confirming convergence to the equilibrium.

### 4.2 Second derivative of Lyapunov function

Considering the model as

$$\frac{d\dot{L}}{dt}=\frac{d}{dt}\left\{\left(\frac{x_{1}-x_{1}^{*}}{x_{1}}\right){\dot{x}}_{1}+\left(\frac{x_{2}-x_{2}^{*}}{x_{2}}\right){\dot{x}}_{2}\right\},$$ (29)

$$\frac{d\dot{L}}{dt}={\left(\frac{{\dot{x}}_{1}}{x_{1}}\right)}^{2}x_{1}^{*}+{\left(\frac{{\dot{x}}_{2}}{x_{2}}\right)}^{2}x_{2}^{*}+\left(1-\frac{x_{1}^{*}}{x_{1}}\right){\ddot{x}}_{1}+\left(x_{2}-\frac{x_{2}^{*}}{x_{2}}\right){\ddot{x}}_{2},$$ (30)

here

$$\begin{array}{ccc} {\ddot{x}}_{1} & = & \alpha_{1}{\dot{x}}_{1}\left(1-\frac{x_{1}}{K_{1}}\right)-\alpha_{1}x_{1}\frac{{\dot{x}}_{1}}{K_{1}}-\beta_{1}{\dot{x}}_{1}x_{2}-\beta_{1}x_{1}{\dot{x}}_{2}-\varepsilon \gamma {\dot{x}}_{1},\\ {\ddot{x}}_{2} & = & \alpha_{2}{\dot{x}}_{2}\left(1-\frac{x_{2}}{K_{2}}\right)-\alpha_{2}x_{2}\frac{{\dot{x}}_{2}}{K_{2}}-\beta_{2}{\dot{x}}_{1}x_{2}-\beta_{2}x_{1}{\dot{x}}_{2}-\gamma {\dot{x}}_{2}, \end{array}$$ (31)

so

$$\begin{array}{ccc} \frac{d\dot{L}}{dt} & = & {\left(\frac{{\dot{x}}_{1}}{x_{1}}\right)}^{2}x_{1}^{*}+{\left(\frac{{\dot{x}}_{2}}{x_{2}}\right)}^{2}x_{2}^{*}\\ & & +\left(1-\frac{x_{1}^{*}}{x_{1}}\right)\left(\alpha_{1}{\dot{x}}_{1}\left(1-\frac{x_{1}}{K_{1}}\right)-\alpha_{1}x_{1}\frac{{\dot{x}}_{1}}{K_{1}}-\beta_{1}{\dot{x}}_{1}x_{2}-\beta_{1}x_{1}{\dot{x}}_{2}-\varepsilon \gamma {\dot{x}}_{1}\right)\\ & & +\left(1-\frac{x_{2}^{*}}{x_{2}}\right)\left(\alpha_{2}{\dot{x}}_{2}\left(1-\frac{x_{2}}{K_{2}}\right)-\alpha_{2}x_{2}\frac{{\dot{x}}_{2}}{K_{2}}-\beta_{2}{\dot{x}}_{1}x_{2}-\beta_{2}x_{1}{\dot{x}}_{2}-\gamma {\dot{x}}_{2}\right). \end{array}$$ (32)

After replacing ${\dot{x}}_{1}$,${\dot{x}}_{2}$, we can acquire by using their formula from the suggested model and putting everything together.

$$\begin{array}{ccc} \frac{d\dot{L}}{dt} & = & \dot{\Pi }(x_{1},x_{2})+\left(1-\frac{x_{1}^{*}}{x_{1}}\right)\left(\alpha_{1}\left(1-\frac{x_{1}}{K_{1}}\right)-\frac{\alpha_{1}x_{1}}{K_{1}}-\beta_{1}x_{2}-\varepsilon \gamma \right)\\ & & \times \left(\alpha_{1}x_{1}\left(1-\frac{x_{1}}{K_{1}}\right)-\beta_{1}x_{1}x_{2}-\gamma \varepsilon x_{1}\right)\\ & & -\left(1-\frac{x_{1}^{*}}{x_{1}}\right)\left(\beta_{1}x_{1}\left(\alpha_{2}x_{2}\left(1-\frac{x_{2}}{K_{2}}\right)-\beta_{2}x_{1}x_{2}-\gamma x_{2}\right)\right)\times \left(\alpha_{1}x_{1}\left(1-\frac{x_{1}}{K_{1}}\right)-\beta_{1}x_{1}x_{2}-\gamma \varepsilon x_{1}\right)\\ & & +\left(1-\frac{x_{2}^{*}}{x_{2}}\right)\left(\alpha_{2}\left(1-\frac{x_{2}}{K_{2}}\right)-\frac{\alpha_{2}x_{2}}{K_{2}}-\beta_{2}x_{1}-\gamma \right)\times \left(\alpha_{2}x_{2}\left(1-\frac{x_{2}}{K_{2}}\right)-\beta_{2}x_{1}x_{2}-\gamma x_{2}\right)\\ & & -\left(1-\frac{x_{2}^{*}}{x_{2}}\right)\left(\beta_{2}x_{2}\left(\alpha_{1}x_{1}\left(1-\frac{x_{1}}{K_{1}}\right)-\beta_{1}x_{1}x_{2}-\gamma \varepsilon x_{1}\right)\right)\times \left(\alpha_{2}x_{2}\left(1-\frac{x_{2}}{K_{2}}\right)-\beta_{2}x_{1}x_{2}-\gamma x_{2}\right). \end{array}$$ (33)

By ensuring that the net growth terms of both healthy and malignant cells satisfy their equilibrium constraints, we can guarantee that


$$\frac{d\dot{L}}{dt}\leq 0.$$

Thus, the system is globally stable at the equilibrium provided:


$$R_{0}=\frac{\alpha_{2}}{\gamma +\beta_{2}K_{1}}<1,\;\frac{\alpha_{1}-\varepsilon \gamma }{\beta_{1}K_{2}}>1.$$

These conditions ensure that cancer cell growth is suppressed while healthy cells outcompete them, leading to system stability.

## 5 Existence and uniqueness results

Using linear growth results, we find the existence and uniqueness of the model (8). Consider the model

$$\begin{array}{ccccc} {\;}^{FFM}D_{0,t}^{\mu ,\nu }x_{1} & = & \alpha_{1}x_{1}-\frac{\alpha_{1}x_{1}^{2}}{K_{1}}-\beta_{1}x_{1}x_{2}-\varepsilon \gamma x_{1}=F_{1}(t,x_{1},x_{2}),{\;}^{FFM}D_{0,t}^{\mu ,\nu }x_{2} & = & \alpha_{2}x_{2}-\frac{\alpha_{2}x_{2}^{2}}{K_{2}}-\beta_{2}x_{1}x_{2}-\gamma x_{2}=F_{2}(t,x_{1},x_{2}). \end{array}$$ (34)

To prove the existence and uniqueness of the solution, we verify the following:

(*i*) ${\left|F(t,x_{i})\right|}^{2}\leq K_{i}\left(1+{\left|x_{i}\right|}^{2}\right),i=1,2.$

(*i*) ${\left|F_{i}(t,x_{i}^{1})-F_{i}(t,x_{i}^{2})\right|}^{2}\leq M_{i}{\left|x_{i}^{1}-x_{i}^{2}\right|}^{2}.$

We start with linear growth:


$${\left|F_{1}(t,x_{1},x_{2})\right|}^{2}={\left|\alpha_{1}x_{1}-\frac{\alpha_{1}x_{1}^{2}}{K_{1}}-\beta_{1}x_{1}x_{2}-\varepsilon \gamma x_{1}\right|}^{2}$$

$$\leq 4\alpha_{1}^{2}{\left|x_{1}\right|}^{2}+4\frac{\alpha_{1}^{2}}{K_{1}^{2}}{\left|x_{1}^{2}\right|}^{2}+4\beta_{1}^{2}{\left|x_{1}\right|}^{2}{\left|x_{2}\right|}^{2}+4\varepsilon^{2}\gamma^{2}{\left|x_{1}\right|}^{2}$$

$$\leq 4\left(\alpha_{1}^{2}+\frac{\alpha_{1}^{2}}{K_{1}^{2}}\sup_{t\in [0,T]}{\left|x_{1}\right|}^{2}+\beta_{1}^{2}\sup_{t\in [0,T]}{\left|x_{2}\right|}^{2}+\varepsilon^{2}\gamma^{2}\right){\left|x_{1}\right|}^{2}$$ (35)

$$\leq 4\left(\alpha_{1}^{2}+\frac{\alpha_{1}^{2}}{K_{1}^{2}}M_{1}+\beta_{1}^{2}M_{2}+\varepsilon^{2}\gamma^{2}\right){\left|x_{1}\right|}^{2}$$

$$\leq 4\left(\alpha_{1}^{2}+\frac{\alpha_{1}^{2}}{K_{1}^{2}}M_{1}+\beta_{1}^{2}M_{2}+\varepsilon^{2}\gamma^{2}\right)\left(1+{\left|x_{1}\right|}^{2}\right)$$

where *M* is such that $\sup_{t\in [0,T]}{\left|x_{1}\right|}^{2}\leq M$ and $\sup_{t\in [0,T]}{\left|x_{2}\right|}^{2}\leq M$. Hence, $\left|F(t,x_{1},x_{2})\right|\leq k_{1}\left(1+{\left|x_{1}\right|}^{2}\right).$ Where $k_{1}=4\left(\alpha_{1}^{2}+\frac{\alpha_{1}^{2}}{K_{1}^{2}}M_{1}+\beta_{1}^{2}M_{2}+\varepsilon^{2}\gamma^{2}\right).$ Similarly , we have


$${\left|F_{2}(t,x_{1},x_{2})\right|}^{2}={\left|\alpha_{2}x_{2}-\frac{\alpha_{2}x_{2}^{2}}{K_{2}}-\beta_{2}x_{1}x_{2}-\gamma x_{2}\right|}^{2}$$

$$\leq 4\alpha_{2}^{2}{\left|x_{2}\right|}^{2}+4\frac{\alpha_{2}^{2}}{K_{2}^{2}}{\left|x_{2}^{2}\right|}^{2}+4\beta_{2}^{2}{\left|x_{1}\right|}^{2}{\left|x_{2}\right|}^{2}+4\gamma^{2}{\left|x_{2}\right|}^{2}$$

$$\leq 4\left(\alpha_{2}^{2}+\frac{\alpha_{2}^{2}}{K_{2}^{2}}\sup_{t\in [0,T]}{\left|x_{2}\right|}^{2}+\beta_{2}^{2}\sup_{t\in [0,T]}{\left|x_{1}\right|}^{2}+\gamma^{2}\right){\left|x_{2}\right|}^{2}$$ (36)

$$\leq 4\left(\alpha_{2}^{2}+\frac{\alpha_{2}^{2}}{K_{2}^{2}}M_{3}+\beta_{2}^{2}M_{4}+\gamma^{2}\right){\left|x_{2}\right|}^{2}$$

$$\leq 4\left(\alpha_{1}^{2}+\frac{\alpha_{2}^{2}}{K_{2}^{2}}M_{3}+\beta_{2}^{2}M_{4}+\gamma^{2}\right)\left(1+{\left|x_{2}\right|}^{2}\right)$$

where $\sup_{t\in [0,T]}{\left|x_{1}\right|}^{2}\leq M,$ and $\sup_{t\in [0,T]}{\left|x_{2}\right|}^{2}\leq M$. Hence $\left|F_{2}(t,x_{1},x_{2})\right|\leq k_{2}\left(1+{\left|x_{2}\right|}^{2}\right).$ Where $k_{2}=4\left(\alpha_{1}^{2}+\frac{\alpha_{2}^{2}}{K_{2}^{2}}M_{3}+\beta_{2}^{2}M_{4}+\gamma^{2}\right).$ Therefore, the function satisfies the growth condition.

Now we will verify the Lipschitz condition:


$${\left|F_{1}(t,x_{1})-F_{1}(t,x_{1}^{1})\right|}^{2}={\left|\alpha_{1}(x_{1}-x_{1}^{1})-\frac{\alpha_{1}(x_{1}^{2}-{\left(x_{1}^{1}\right)}^{2})}{K_{1}}-\beta_{1}(x_{1}-x_{1}^{1})x_{2}-\varepsilon \gamma (x_{1}-x_{1}^{1})\right|}^{2}$$

$$<4\alpha_{1}^{2}{\left|x_{1}-x_{1}^{1}\right|}^{2}+4\frac{\alpha_{1}^{2}}{K_{1}^{2}}{\left|x_{1}^{2}-{\left(x_{1}^{1}\right)}^{2}\right|}^{2}+4\beta_{1}^{2}{\left|x_{1}-x_{1}^{1}\right|}^{2}{\left|x_{2}\right|}^{2}+4\varepsilon^{2}\gamma^{2}{\left|x_{1}-x_{1}^{1}\right|}^{2}$$

$$<4\alpha_{1}^{2}{\left|x_{1}-x_{1}^{1}\right|}^{2}+4\frac{\alpha_{1}^{2}}{K_{1}^{2}}{\left|x_{1}-x_{1}^{1}\right|}^{2}\left(2{\left|x_{1}\right|}^{2}+2{\left|x_{1}^{1}\right|}^{2}\right)+4\beta_{1}^{2}{\left|x_{1}-x_{1}^{1}\right|}^{2}{\left|x_{2}\right|}^{2}+4\varepsilon^{2}\gamma^{2}{\left|x_{1}-x_{1}^{1}\right|}^{2}$$

$$<4\alpha_{1}^{2}{\left|x_{1}-x_{1}^{1}\right|}^{2}+4\frac{\alpha_{1}^{2}}{K_{1}^{2}}{\left|x_{1}-x_{1}^{1}\right|}^{2}\left(2\sup_{t\in [0,T]}{\left|x_{1}\right|}^{2}+2\sup_{t\in [0,T]}{\left|x_{1}^{1}\right|}^{2}\right)$$

$$+4\beta_{1}^{2}{\left|x_{1}-x_{1}^{1}\right|}^{2}\sup_{t\in [0,T]}{\left|x_{2}\right|}^{2}+4\varepsilon^{2}\gamma^{2}{\left|x_{1}-x_{1}^{1}\right|}^{2}$$

$$<\left(4\alpha_{1}^{2}+4\frac{\alpha_{1}^{2}}{K_{1}^{2}}\left(2\sup_{t}{\left|x_{1}\right|}^{2}+2\sup_{t}{\left|x_{1}^{1}\right|}^{2}\right)+4\beta_{1}^{2}\sup_{t\in [0,T]}{\left|x_{2}\right|}^{2}+4\varepsilon^{2}\gamma^{2}\right){\left|x_{1}-x_{1}^{1}\right|}^{2}$$

$$<\left(4\alpha_{1}^{2}+4\frac{\alpha_{1}^{2}}{K_{1}^{2}}\left(2M_{5}+2M_{6}\right)+4\beta_{1}^{2}M_{7}+4\varepsilon^{2}\gamma^{2}\right){\left|x_{1}-x_{1}^{1}\right|}^{2}$$

$$<{\bar{k}}_{1}{\left|x_{1}-x_{1}^{1}\right|}^{2}$$

where ${\bar{k}}_{1}=\left(4\alpha_{1}^{2}+4\frac{\alpha_{1}^{2}}{K_{1}^{2}}\left(2M_{5}+2M_{6}\right)+4\beta_{1}^{2}M_{7}+4\varepsilon^{2}\gamma^{2}\right)$ and assume that $\sup_{t\in [0,T]}{\left|x_{1}\right|}^{2}\leq M,$ and $\sup_{t\in [0,T]}{\left|x_{2}\right|}^{2}\leq M$. Similarly, we have


$${\left|F_{2}(t,x_{2})-F_{1}(t,x_{2}^{1})\right|}^{2}={\left|\alpha_{2}(x_{2}-x_{2}^{1})-\frac{\alpha_{2}(x_{2}^{2}-{\left(x_{2}^{1}\right)}^{2})}{K_{2}}-\beta_{2}(x_{2}-x_{2}^{1})x_{1}-\gamma (x_{2}-x_{2}^{1})\right|}^{2}$$

$$<4\alpha_{2}^{2}{\left|x_{2}-x_{2}^{1}\right|}^{2}+4\frac{\alpha_{2}^{2}}{K_{2}^{2}}{\left|x_{2}^{2}-{\left(x_{2}^{1}\right)}^{2}\right|}^{2}+4\beta_{2}^{2}{\left|x_{2}-x_{2}^{1}\right|}^{2}{\left|x_{1}\right|}^{2}+4\gamma^{2}{\left|x_{2}-x_{2}^{1}\right|}^{2}$$

$$<4\alpha_{2}^{2}{\left|x_{2}-x_{2}^{1}\right|}^{2}+4\frac{\alpha_{2}^{2}}{K_{2}^{2}}{\left|x_{2}-x_{2}^{1}\right|}^{2}\left(2{\left|x_{2}\right|}^{2}+2{\left|x_{2}^{1}\right|}^{2}\right)+4\beta_{2}^{2}{\left|x_{2}-x_{2}^{1}\right|}^{2}{\left|x_{1}\right|}^{2}+4\gamma^{2}{\left|x_{2}-x_{2}^{1}\right|}^{2}$$

$$<4\alpha_{2}^{2}{\left|x_{2}-x_{2}^{1}\right|}^{2}+4\frac{\alpha_{2}^{2}}{K_{2}^{2}}{\left|x_{2}-x_{2}^{1}\right|}^{2}\left(2\sup_{t\in [0,T]}{\left|x_{2}\right|}^{2}+2\sup_{t\in [0,T]}{\left|x_{2}^{1}\right|}^{2}\right)$$

$$+4\beta_{2}^{2}{\left|x_{2}-x_{2}^{1}\right|}^{2}\sup_{t\in [0,T]}{\left|x_{1}\right|}^{2}+4\gamma^{2}{\left|x_{2}-x_{2}^{1}\right|}^{2}$$

$$<\left(4\alpha_{2}^{2}+4\frac{\alpha_{2}^{2}}{K_{2}^{2}}\left(2\sup_{t\in [0,T]}{\left|x_{2}\right|}^{2}+2\sup_{t\in [0,T]}{\left|x_{2}^{1}\right|}^{2}\right)+4\beta_{2}^{2}\sup_{t}{\left|x_{1}\right|}^{2}+4\gamma^{2}\right){\left|x_{2}-x_{2}^{1}\right|}^{2}$$

$$<\left(4\alpha_{2}^{2}+4\frac{\alpha_{2}^{2}}{K_{2}^{2}}\left(2M_{8}+2M_{9}\right)+4\beta_{2}^{2}M_{10}+4\gamma^{2}\right){\left|x_{2}-x_{2}^{1}\right|}^{2}$$

$$<{\bar{k}}_{2}{\left|x_{2}-x_{2}^{1}\right|}^{2}$$

$${\bar{k}}_{2}=\left(4\alpha_{2}^{2}+4\frac{\alpha_{2}^{2}}{K_{2}^{2}}\left(2M_{8}+2M_{9}\right)+4\beta_{2}^{2}M_{10}+4\gamma^{2}\right).$$

where ${\bar{k}}_{2}=\left(4\alpha_{2}^{2}+4\frac{\alpha_{2}^{2}}{K_{2}^{2}}\left(2M_{8}+2M_{9}\right)+4\beta_{2}^{2}M_{10}+4\gamma^{2}\right)$ and assume that $\sup_{t\in [0,T]}{\left|x_{1}\right|}^{2}\leq M,$ and

$\sup_{t\in [0,T]}{\left|x_{2}\right|}^{2}\leq M$. Under these conditions, the system has a unique solution.

**Remarks 4.** The necessary conditions for the existence of a unique solution to the proposed fractal-fractional cancer treatment model have been thoroughly established. These conditions include the continuity and Lipschitz properties of the functions involved, the provision of appropriate initial conditions that ensure positivity, and the adherence to linear growth constraints. By applying fixed-point theory, the authors rigorously demonstrate that these conditions are satisfied, thus ensuring the reliability of the model in simulating tumor dynamics under various treatment scenarios. This foundational work plays a crucial role in validating subsequent analyses concerning the stability of the system and the effectiveness of treatment strategies within the proposed mathematical framework.

## 6 Numerical scheme

Here, we define a system (8) operator with a fractal fractional for piecewise Lagrange interpolation [53]. The problem (8) is further solved by applying the fractal fraction integral. Thus


$$\begin{array}{ccc} x_{1}(t) & = & x_{1}(0)+\frac{\nu t^{\nu -1}(1-\mu )}{AB(\mu )}A_{1}(t,x_{1},x_{2})+\frac{\mu \nu_{1}}{AB(\mu )\Gamma (\mu )}\\ & & \times \int_{0}^{t}\sigma^{\nu -1}(t-\sigma {)}^{\mu -1}A_{1}(t,x_{1},x_{2})d\sigma , \end{array}$$

$$\begin{array}{ccc} x_{2}(t) & = & x_{2}(0)+\frac{\nu t^{\nu -1}(1-\mu )}{AB(\mu )}A_{2}(t,x_{1},x_{2})+\frac{\mu \nu }{AB(\mu )\Gamma (\mu )}\\ & & \times \int_{0}^{t}\sigma^{\nu -1}(t-\sigma {)}^{\mu -1}A_{2}(t,x_{1},x_{2})d\sigma , \end{array}$$ (37)

The numerical scheme is now derived at *t* = *t*_*r* + 1_, r=0,1,2,..., we have

$$\left\{ \begin{array}{c} x_{1}^{r+1}=x_{1}^{0}+\frac{\nu t^{\nu -1}(1-\mu )}{AB(\mu )}A_{1}(t^{r},x_{1}^{r},x_{2}^{r})+\frac{\mu \nu }{AB(\mu )\Gamma (\mu )}\\ \times \int_{0}^{t}\sigma^{\nu -1}(t-\sigma {)}^{\mu -1}A_{1}(t,x_{1},x_{2})d\sigma ,\\ x_{2}^{r+1}=x_{2}^{0}+\frac{\nu t^{\nu -1}(1-\mu )}{AB(\mu )}A_{2}(t^{r},x_{1}^{r},x_{2}^{r})+\frac{\mu \nu }{AB(\mu )\Gamma (\mu )}\\ \times \int_{0}^{t}\sigma^{\nu -1}(t-\sigma {)}^{\mu -1}A_{2}(t,x_{1},x_{2})d\sigma . \end{array}\right.$$ (38)

When we approximate the integrals on the right side of the system (38), we get

$$\left\{ \begin{array}{c} x_{1}^{r+1}=x_{1}^{0}+\frac{\nu t^{\nu -1}(1-\mu )}{AB(\mu )}A_{1}(t^{r},x_{1}^{r},x_{2}^{r})+\frac{\mu \nu }{AB(\mu )\Gamma (\mu )}\\ \times \sum_{v=0}^{r}\int_{t_{v}}^{t_{v+1}}\sigma^{\nu -1}{\left(t_{r+1}-\sigma \right)}^{\mu -1}A_{1}(t,x_{1},x_{2})d\sigma ,\\ x_{2}^{r+1}=x_{2}^{0}+\frac{\nu t^{\nu -1}(1-\mu )}{AB(\mu )}A_{2}(t^{r},x_{1}^{r},x_{2}^{r})+\frac{\mu \nu }{AB(\mu )\Gamma (\mu )}\\ \times \sum_{v=0}^{r}\int_{t_{v}}^{t_{v+1}}\sigma^{\nu -1}{\left(t_{r+1}-\sigma \right)}^{\mu -1}A_{2}(t,x_{1},x_{2})d\sigma ,. \end{array}\right.$$ (39)

Within the finite interval, using Lagrangian piece-wise interpolation $\left[t_{v},t_{v+1}\right]$, we approximate the kernel inside the integrals like this:

$$\left\{ \begin{array}{c} x_{1}(\sigma )=\frac{\sigma -t_{v-1}}{t_{v}-t_{v-1}}t_{v}^{{\;}^{\nu -1}}A_{1}(t^{v},x_{1}^{v},x_{2}^{v})-\frac{\sigma -t_{v}}{t_{v}-t_{v-1}}t_{v-1}^{{\;}^{\nu -1}}\\ \times A_{1}(t^{v-1},x_{1}^{v-1},x_{2}^{v-1}),\\ x_{2}(\sigma )=\frac{\sigma -t_{v-1}}{t_{v}-t_{v-1}}t_{v}^{{\;}^{\nu -1}}A_{2}(t^{v},x_{1}^{v},x_{2}^{v})-\frac{\sigma -t_{v}}{t_{v}-t_{v-1}}t_{v-1}^{{\;}^{\nu -1}}\\ \times A_{2}(t^{v-1},x_{1}^{v-1},x_{2}^{v-1}). \end{array}\right.$$ (40)

After simplification, we get

$$\left\{ \begin{array}{c} x_{1}^{r+1}=x_{1}^{0}+\frac{\nu t_{r}^{\nu -1}(1-\mu )}{AB(\mu )}A_{1}(t^{r},x_{1}^{r},x_{2}^{r})+\frac{\mu \nu {\left(\Delta t\right)}^{\mu }}{AB(\mu )\Gamma (\mu +2)}\\ \times \sum_{v=0}^{r}\left[\begin{array}{c} t_{v}^{\nu -1}A_{1}(t^{v},x_{1}^{v},x_{2}^{v})\times (q^{\mu }(q+1+\mu )\\ -p^{\mu }(1+2\mu +q))-t_{v-1}^{\nu -1}A_{1}(t^{v-1},x_{1}^{v-1},x_{2}^{v-1})\\ \times (q^{\mu +1}-p^{\mu }(q+\mu )) \end{array}\right]\\ x_{2}^{r+1}=x_{2}^{0}+\frac{\nu t_{r}^{\nu -1}(1-\mu )}{AB(\mu )}A_{2}(t^{r},x_{1}^{r},x_{2}^{r})+\frac{\mu \nu {\left(\Delta t\right)}^{\mu }}{AB(\mu )\Gamma (\mu +2)}\\ \times \sum_{v=0}^{r}\left[\begin{array}{c} t_{v}^{\nu -1}A_{2}(t^{v},x_{1}^{v},x_{2}^{v})\times (q^{\mu }(q+1+\mu )\\ -(p^{\mu }(1+2\mu +q))-t_{v-1}^{\nu -1}A_{2}(t^{v-1},x_{1}^{v-1},x_{2}^{v-1})\\ \times (q^{\mu +1}-p^{\mu }(\mu +q)) \end{array}\right] \end{array}\right.$$ (41)

where

r+1−v=q and r−v=p.

## 7 Results of proposed model

**Fig 1:**
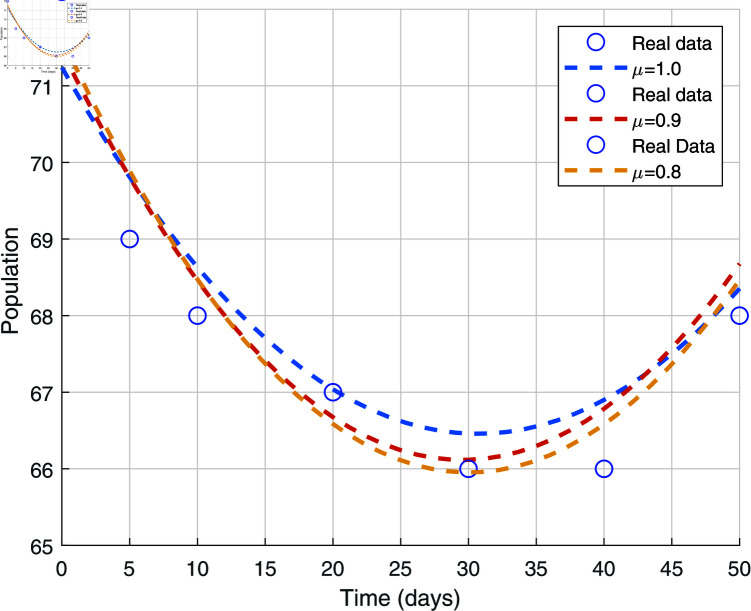
Data fitting using the model (8)

We use the least squares approach to fit curves to evaluate our model and make sure that the results match the real data. We used the actual data for various fractional-order values in Fig 1.

**Fig 2:**
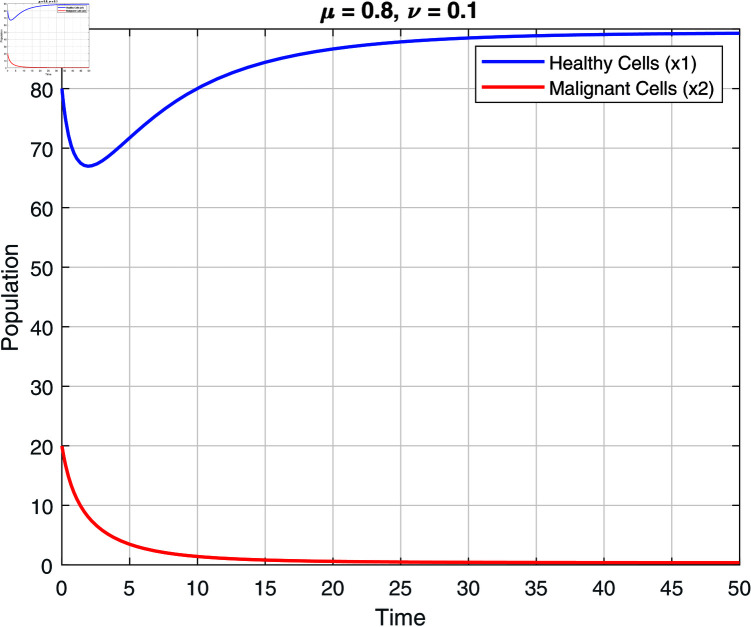
Stability of model (8) at μ=0.8 and ν=0.1.

**Fig 3:**
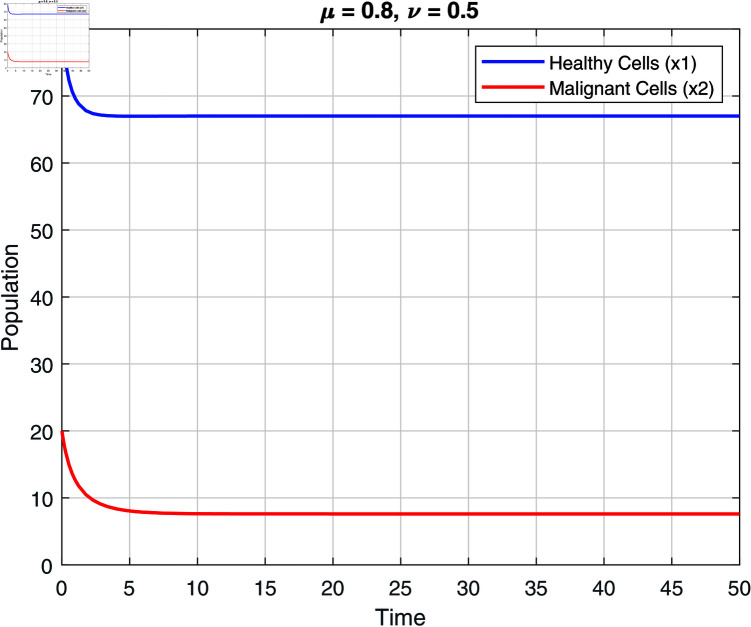
Stability of model (8) at μ=0.8 and ν=0.5.

**Fig 4:**
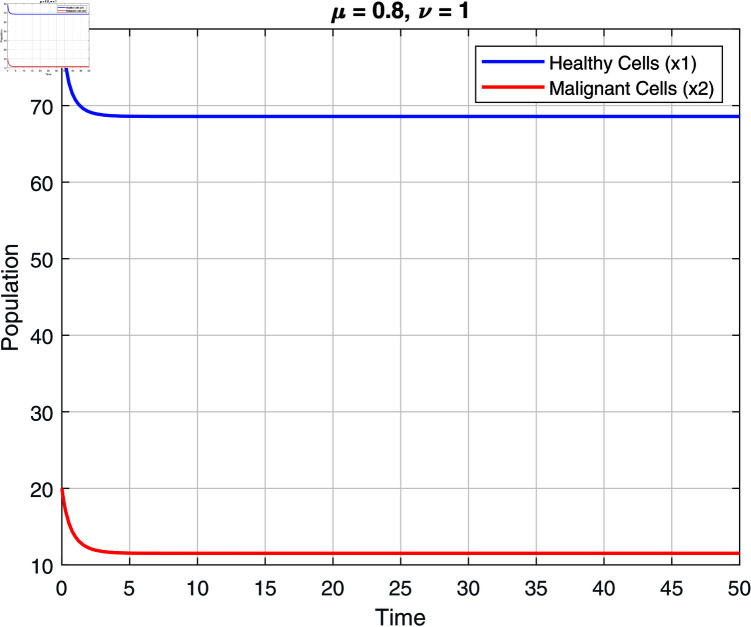
Stability of model (8) at μ=0.8 and ν=1.

**Fig 5:**
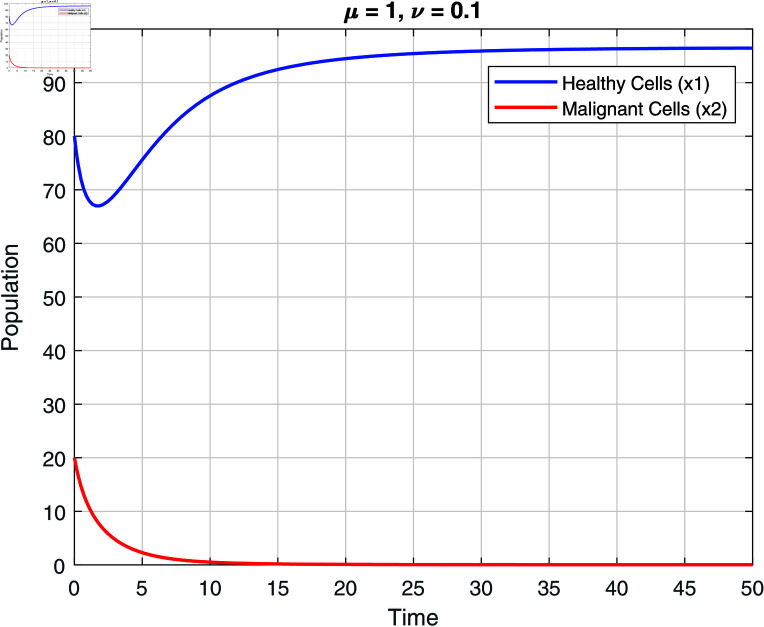
Stability of model (8) at μ=1 and ν=0.1.

**Fig 6:**
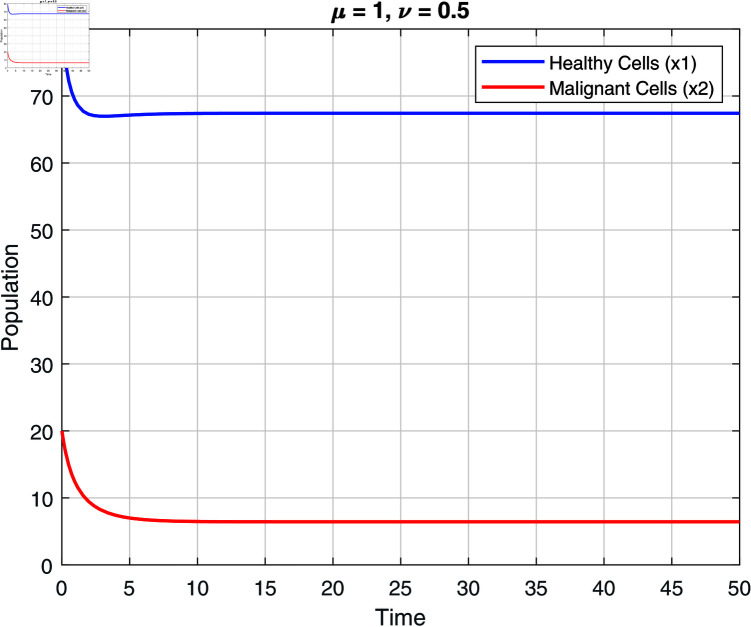
Stability of model (8) at μ=1 and ν=0.5.

**Fig 7:**
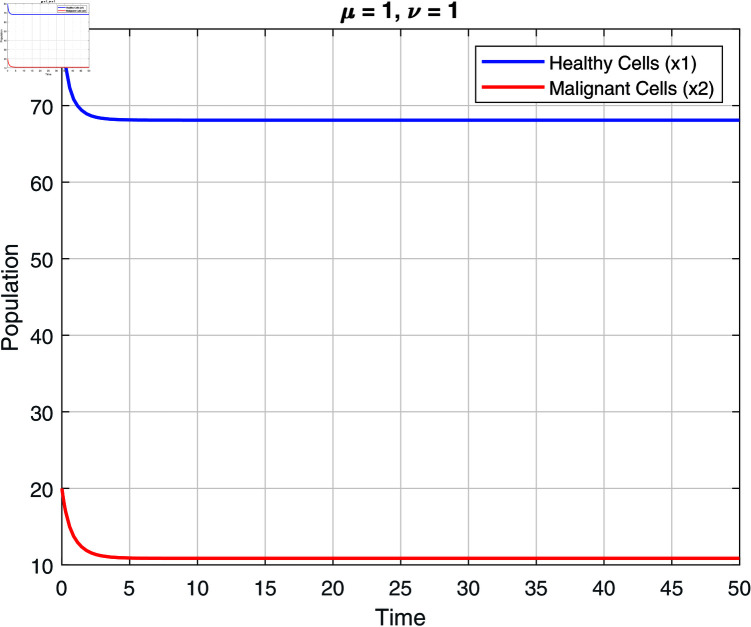
Stability of model (8) at μ=1 and ν=1.

We note that the fractional orders μ and ν have an impact on the stability zones. Regions of stability with respect to μ and ν are displayed in Figs 2–7. The stability domain grows when μ and ν values fall, as seen in Figs 2–7

To demonstrate the accuracy of the fractal fractional solution, we provide numerical examples in this section. With three patients, we performed numerical simulations of the model. Initial values in this study are established based on empirical data from prior research, ensuring they reflect realistic biological conditions. The initial conditions and parameter values listed in the following table were taken from [7, 40], which are given in Table 1. For fractional order μ=0.75, μ=0.85, μ=0.95 and μ=1 and fractal dimension ν=1, Tables 2–7 show approximations of normal cell and cancer cell of the model (8) for three patients. To estimate approximations of the solutions for the cancer mathematical models, it has been found that the fractal fractional operator is quite accurate and efficient.

**Table 1:** Table of parameters and initial values [7, 40].

Radiation	Unit	Patient 1	Patient 1	Patient 2	Patient 2	Patient 3	Patient 3
Parameters							
$\alpha_{1}$	${\text{hour}}^{-1}$	$9.7041\times 10^{-4}$	$9.7041\times 10^{-4}$	$9.7041\times 10^{-4}$	$9.7041\times 10^{-4}$	$9.7041\times 10^{-4}$	$9.7041\times 10^{-4}$
α2	${\text{hour}}^{-1}$	0.3396	0.3396	0.3396	0.3396	0.3396	0.3396
$\beta_{1}$	${\text{hour}}^{-1}$	0.0433	0.0433	0.0433	0.0433	0.0433	0.0433
$\beta_{2}$	${\text{hour}}^{-1}$	0.2385	0.2385	0.2385	0.2385	0.2385	0.2385
*K*_1_	cells	1	1	1	1	1	1
*K*_2_	cells	1	1	1	1	1	1
ϵ	dimensionless	0.0008	0.0008	0.0008	0.0008	0.0008	0.0008
γ	$\frac{\text{Gray}}{\text{hour}}$	0.75	0.35	0.65	0.35	0.85	0.35
Initial *x*_1_	cells	0.284	0.284	0.174	0.174	0.306	0.306
Initial *x*_2_	cells	0.284	0.284	0.174	0.174	0.306	0.306

**Table 2:** Table of patient 1 normal cell at ν=1.

t	μ=0.75	μ=0.85	μ=0.95	μ=1
50	0.2625	0.261	0.2608	0.2614
100	0.2604	0.2606	0.2636	0.2666
150	0.2601	0.2619	0.2676	0.2724
200	0.2603	0.2637	0.2718	0.2782
250	0.2608	0.2656	0.276	0.2841
300	0.2615	0.2676	0.2803	0.29
350	0.2622	0.2697	0.2846	0.296
400	0.263	0.2718	0.2888	0.3019

**Table 3:** Table of patient 1 cancer cell at ν=1.

t	μ=0.75	μ=0.85	μ=0.95	μ=1
50	0.0532	0.0311	0.0118	0.0036
100	0.0324	0.0151	0.0036	0.0001
150	0.0238	0.0099	0.0021	0.0000
200	0.019	0.0074	0.0014	0.0000
250	0.016	0.0059	0.0011	0.0000
300	0.0138	0.0049	0.0009	0.0000
350	0.0122	0.0043	0.0008	0.0000
400	0.011	0.0037	0.0007	0.0000

**Table 4:** Table of patient 2 normal cell at ν=1.

t	μ=0.75	μ=0.85	μ=0.95	μ=1
50	0.1641	0.1631	0.1626	0.1627
100	0.163	0.1629	0.1645	0.1661
150	0.1629	0.164	0.1674	0.1704
200	0.1632	0.1653	0.1707	0.1749
250	0.1636	0.1669	0.174	0.1795
300	0.1642	0.1685	0.1773	0.1842
350	0.1648	0.1701	0.1807	0.189
400	0.1655	0.1717	0.1841	0.1938

**Table 5:** Table of patient 2 cancer cell at ν=1.

t	μ=0.75	μ=0.85	μ=0.95	μ=1
50	0.0463	0.0302	0.0148	0.0076
100	0.0293	0.0151	0.0044	0.0006
150	0.0218	0.0098	0.0022	0.0000
200	0.0176	0.0072	0.0014	0.0000
250	0.0148	0.0057	0.0011	0.0000
300	0.0128	0.0047	0.0009	0.0000
350	0.0113	0.004	0.0007	0.0000
400	0.0101	0.0035	0.0006	0.0000

**Table 6:** Table of patient 3 normal cell at ν=1.

t	μ=0.75	μ=0.85	μ=0.95	μ=1
50	0.282	0.2804	0.2803	0.2811
100	0.2797	0.2799	0.2833	0.2865
150	0.2792	0.2812	0.2873	0.2924
200	0.2794	0.283	0.2916	0.2984
250	0.2799	0.285	0.2959	0.3044
300	0.2806	0.2871	0.3003	0.3104
350	0.2814	0.2891	0.3046	0.3165
400	0.2822	0.2913	0.309	0.3225

**Table 7:** Table of patient 3 cancer cell at ν=1.

t	μ=0.75	μ=0.85	μ=0.95	μ=1
50	0.0541	0.0311	0.0113	0.003
100	0.0328	0.0151	0.0035	0.0001
150	0.0241	0.0099	0.002	0.0000
200	0.0192	0.0074	0.0014	0.0000
250	0.0161	0.006	0.0011	0.0000
300	0.014	0.005	0.0009	0.0000
350	0.0124	0.0043	0.0008	0.0000
400	0.0111	0.0038	0.0007	0.0000

**Fig 8:**
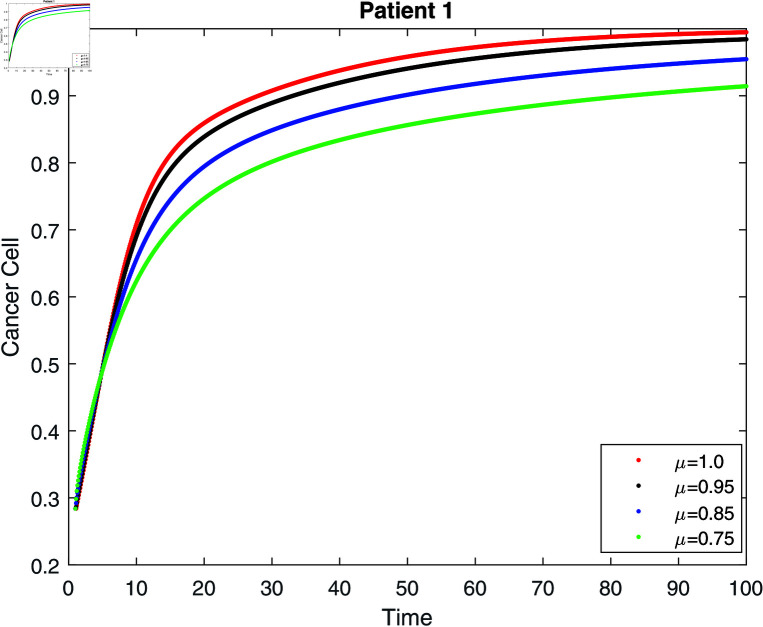
Simulation of patient 1 for cancer cell without treatment at fractal fractional dimension 1.

**Fig 9:**
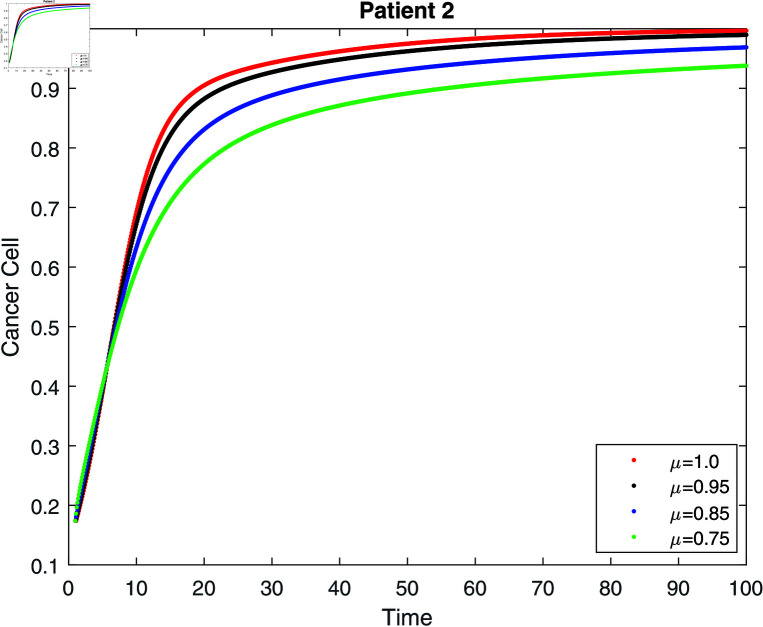
Simulation of patient 2 for cancer cell without treatment at fractal fractional dimension 1.

**Fig 10:**
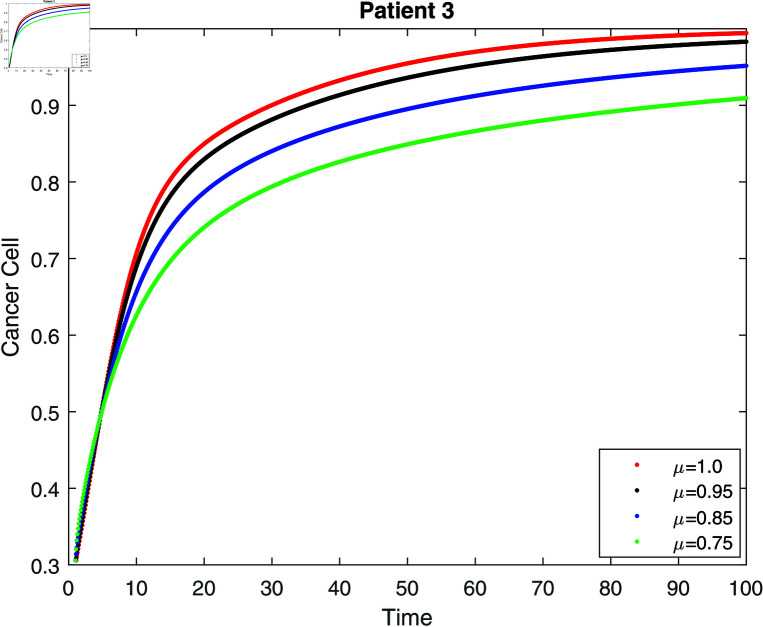
Simulation of patient 3 for cancer cell without treatment at fractal fractional dimension 1.

If we do not receive treatment, the tumor will continue to grow until it exceeds its carrying capacity (γ=0) (see Figs 8–10. According to [6, 31], the population reaches its carrying capacity earlier than anticipated in the classical model. The lower the order of the derivative, however, the slower the convergence to the carrying capacity, which is one of the benefits of the fractional model. Then, for three patients, we give six simulations (see Table 1).

### 7.1 Simulation 1 (S1)

**Fig 11:**
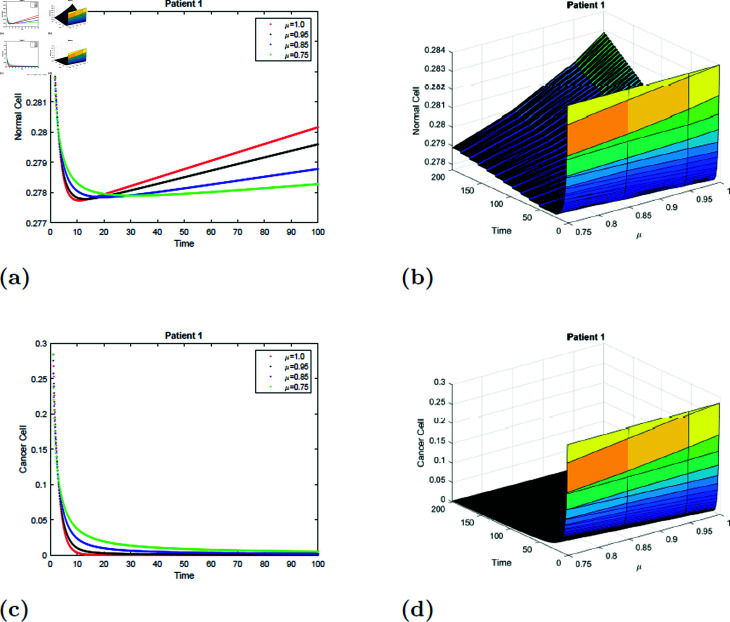
Simulation of Patient 1 at fractal fractional dimension 1 and γ=0.75.

**Fig 12:**
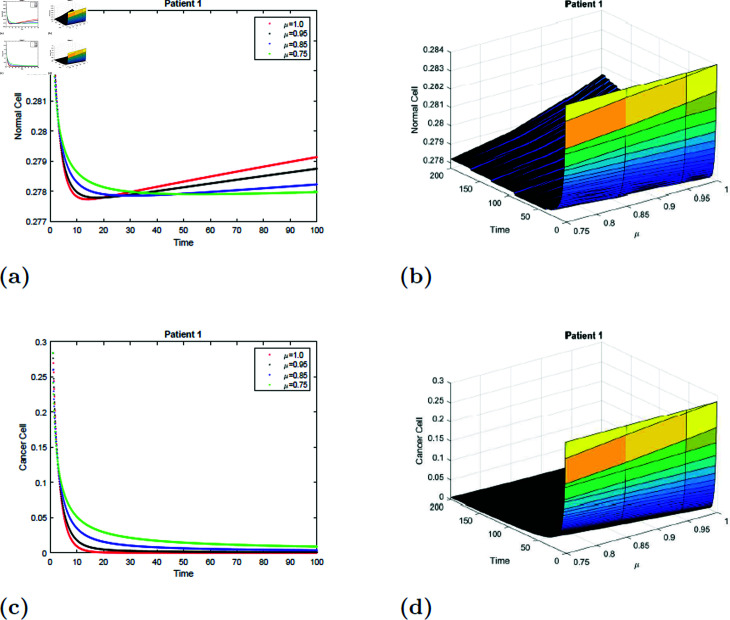
Simulation of Patient 1 at fractal fractional dimension 0.9 and γ=0.75.

With the parameters of *S*1 given in Table 1, the *E*_0_ equilibrium point is stable, as it satisfies Theorem 4.2. Figs 11 and 12 depicted the simulation of the proposed model for patient 1 with fractional order μ=0.75, μ=0.85, μ=0.95 and μ=1 and fractal dimension ν=1. From Figs 11(a) and 12(a), it is observed that initially, the normal cell decreases for all values of μafter20 hours, and the normal cell increases for all values of μ and converges to equilibrium point *E*. The normal cell converges rapidly as the fractional order increases. Figs 11(c) and 12(c) depicted that cancer cells decrease with time for all values of μ. Figs 11(b), 11(d), 12(b), and 12(d) show the surface simulation of normal and cancer cells of patient 1.

### 7.2 Simulation 2 (S2)

**Fig 13:**
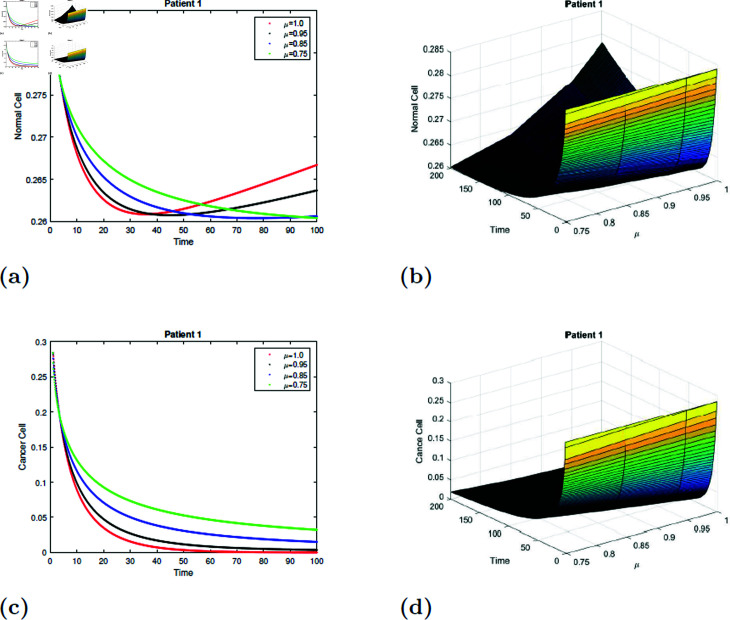
Simulation of Patient 1 at fractal fractional dimension 1 and γ=0.35.

**Fig 14:**
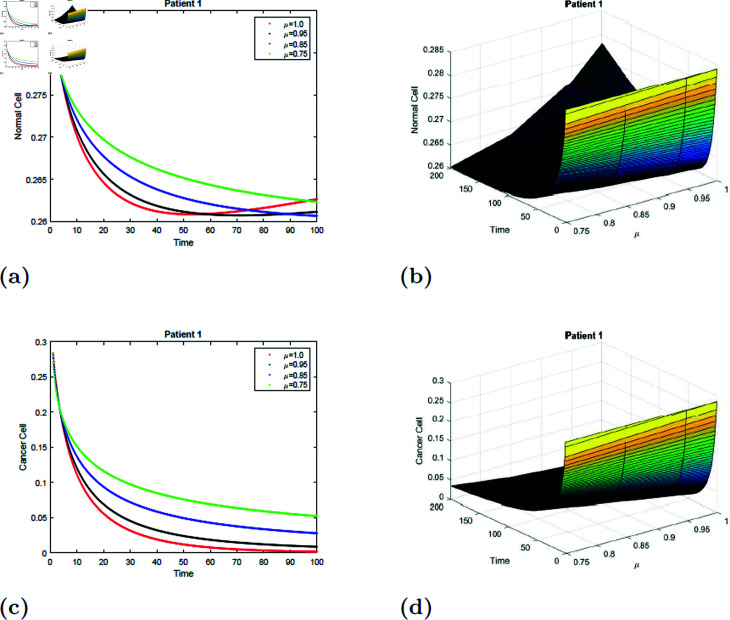
Simulation of Patient 1 at fractal fractional dimension 0.9 and γ=0.35.

The simulation of the suggested model for patient 1 with fractional orders of μ=0.75, μ=0.85, μ=0.95, and μ=1 with a fractal dimension of ν=0.9 is shown in Figs 13 and 14. From Figs 13(a) and 14(a), note that when radiation treatment time increases, the normal cell increases for all values of μ and converges to equilibrium point *E*_0_. Initially, the normal cell decreases for all values of μ. The cancer cell decrease was shown in Figs 13(c) and 14(c) for all values of μ. The surface simulation of the normal and cancer cells of patient 2 is shown in Figs 13(b), 13(d), 14(b), and 14(d). Figs 13 and 14 observe that all other parameters are if nothing changes, the rate of radiation dose increases will rapidly eradicate the cancer cells and increase the simultaneous concentration of the healthy cells.

### 7.3 Simulation 3 (S3)

**Fig 15:**
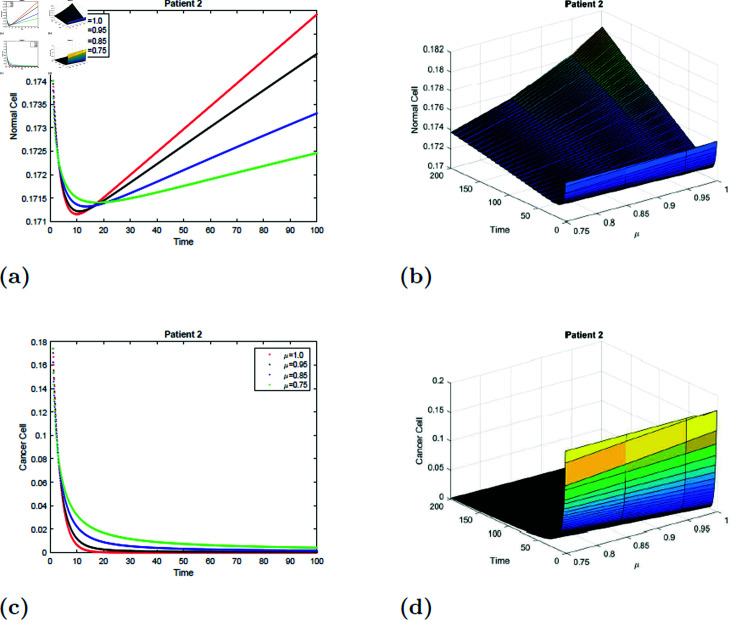
Simulation of Patient 2 at fractal fractional dimension 1 and γ=0.65.

**Fig 16:**
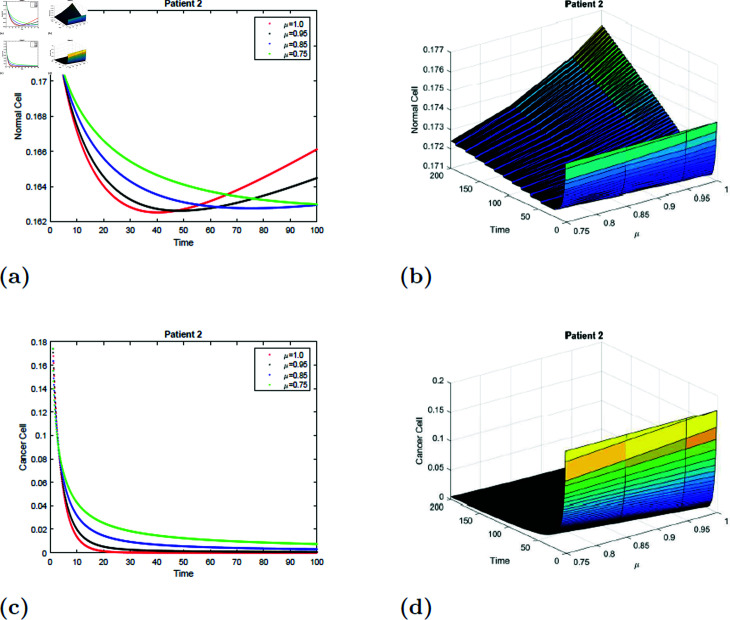
Simulation of Patient 2 at fractal fractional dimension 0.9 and γ=0.65.

With the parameters of S3 given in Table 1, the *E*_0_ equilibrium point is stable, as it satisfies Theorem 4.2. Figs 15 and 16show a simulation of the suggested model for Patient 2 with fractional orders of μ=0.75, μ=0.85, μ=0.95, and μ=1, and fractal dimensions of ν=1. According to Figs 15(a) and 16(a), the normal cell initially decreased for all values of μ; however, increases with time, the normal cell increased for all values of μ and converged to the equilibrium point *E*0. For all values of μ, the Figs 15(c) and 16(c) showed how cancer cells decreased over time. The surface simulation of patient 2’s normal and the cancer cell is depicted in Figs 15(b), 15(d), 16(b), and 16(d).

### 7.4 Simulation 4 (S4)

**Fig 17:**
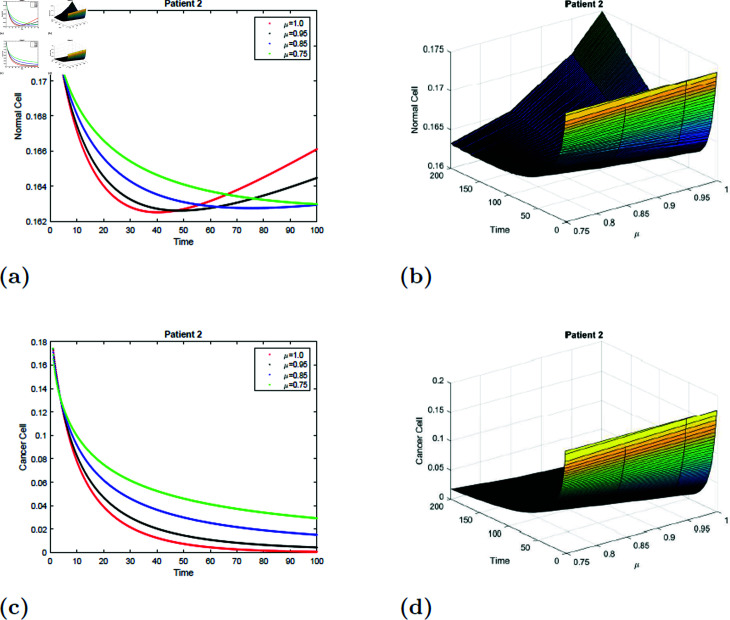
Simulation of Patient 2 at fractal fractional dimension 1 and γ=0.35.

**Fig 18:**
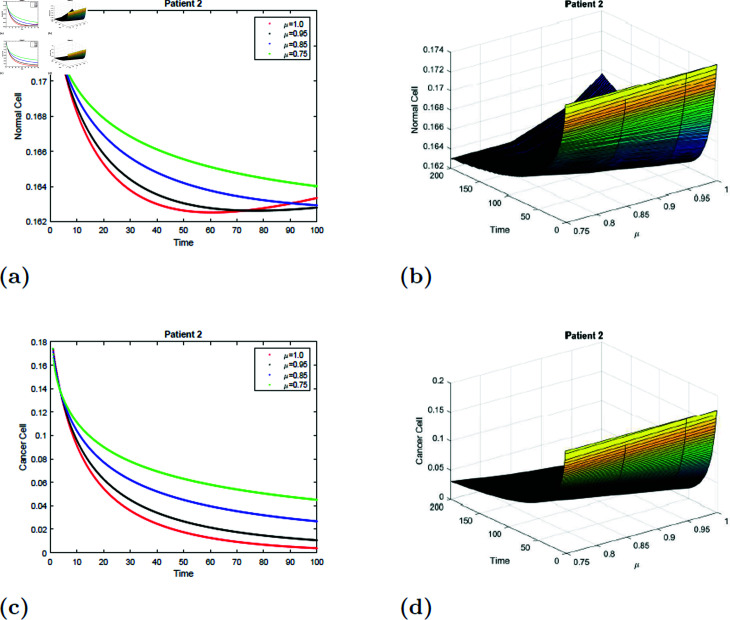
Simulation of Patient 2 at fractal fractional dimension 0.9 and γ=0.35.

The simulation of the suggested model for patient 2 with fractional orders of μ=0.75, μ=0.85, μ=0.95, and *mu* = 1 with a fractal dimension of ν=0.9 is shown in Figs 17 and 18. From Figs 17(a) and 18(a), you can see that after 20 hours, the normal cell increases for all values of *mu* and converges to the equilibrium point *E*_0_. Initially, the normal cell decreases for all values of μ. The cancer cell decline with time was shown in Figs 17(c) and 18(c) for all values of μ. The surface simulation of a normal and cancer cell from patient 2 is shown in Figs 17(b), 17(d), 18(b), and 18(d). Figs 15-18 demonstrate that, if all other parameters remain unchanged, an increase in radiation dosage will speed up the eradication of cancer cells.

### 7.5 Simulation 5 (S5)

**Fig 19:**
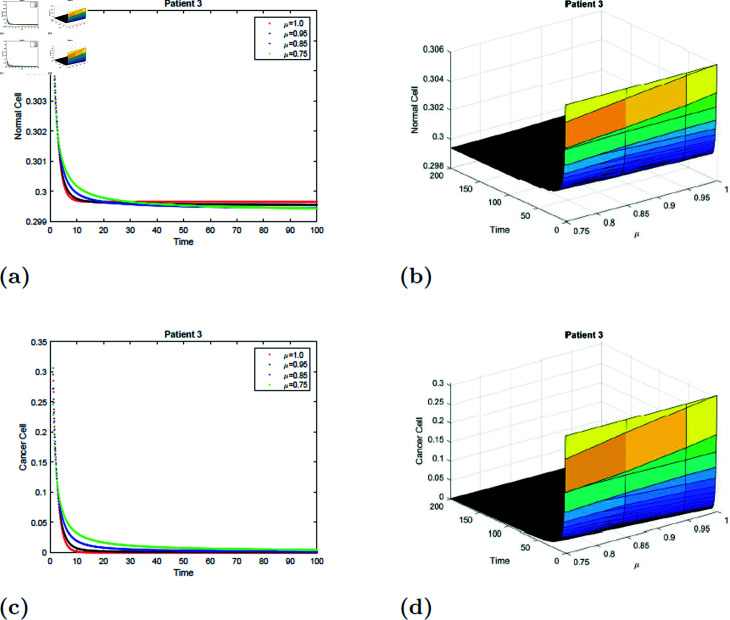
Simulation of Patient 3 at fractal fractional dimension 1 and γ=0.85.

**Fig 20:**
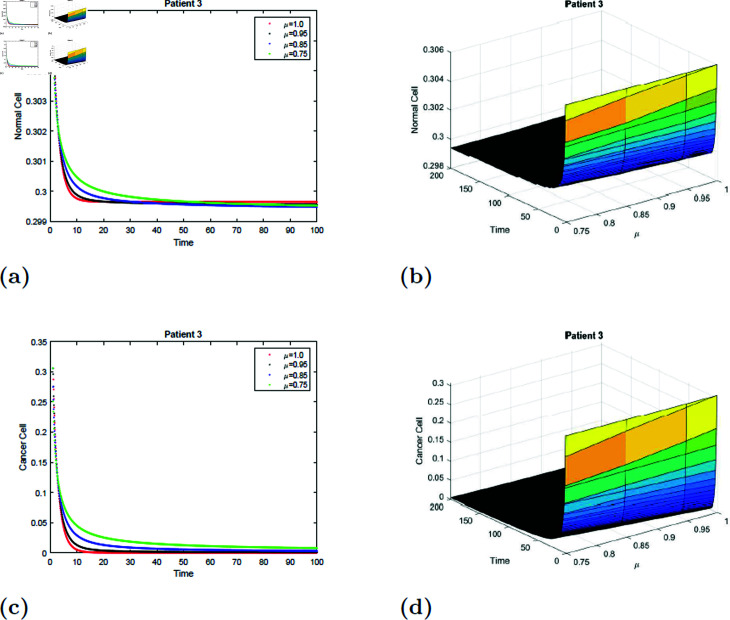
Simulation of Patient 3 at fractal fractional dimension 0.9 and γ=0.85.

With the parameters of S5 given in Table 1, the *E*_0_ equilibrium point is stable, as it satisfies Theorem 4.2. The simulation of the suggested model for patient 3 with fractional orders of μ=0.75, μ=0.85, μ=0.95, and μ=1 with a fractal dimension of ν=1 is shown in Figs 19 and 20. From Figs 19(a) and 20(a), you can see that increasing the radiation time, the normal cell increases for all values of μ and converges to the equilibrium point *E*_0_. Initially, the normal cell decreases for all values of μ. The cancer cell decline with time was shown in Figs 19(c) and 20(c) for all values of μ. The surface simulation of the patient’s 1 normal and cancer cell is shown in Figs 19(b), 19(d), 20(b), and 20(d).

### 7.6 Simulation 6 (S6)

**Fig 21:**
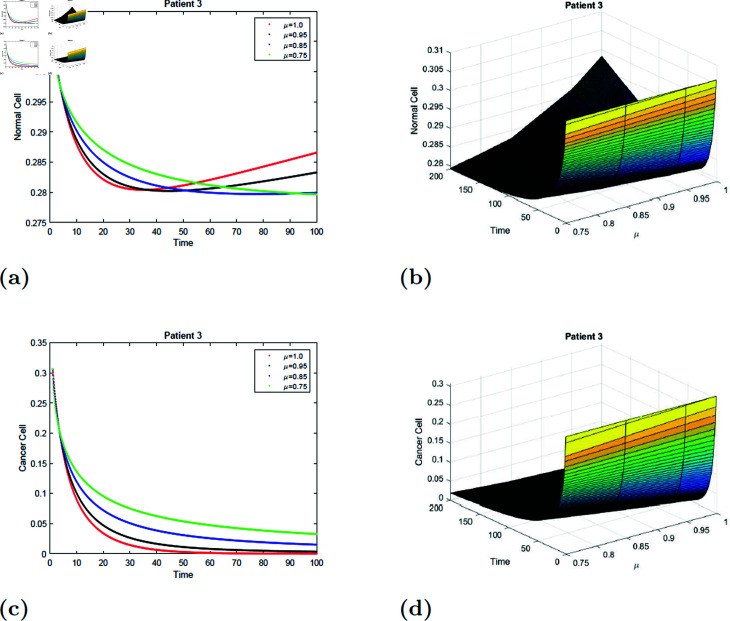
Simulation of Patient 3 at fractal fractional dimension 1 and γ=0.35.

**Fig 22:**
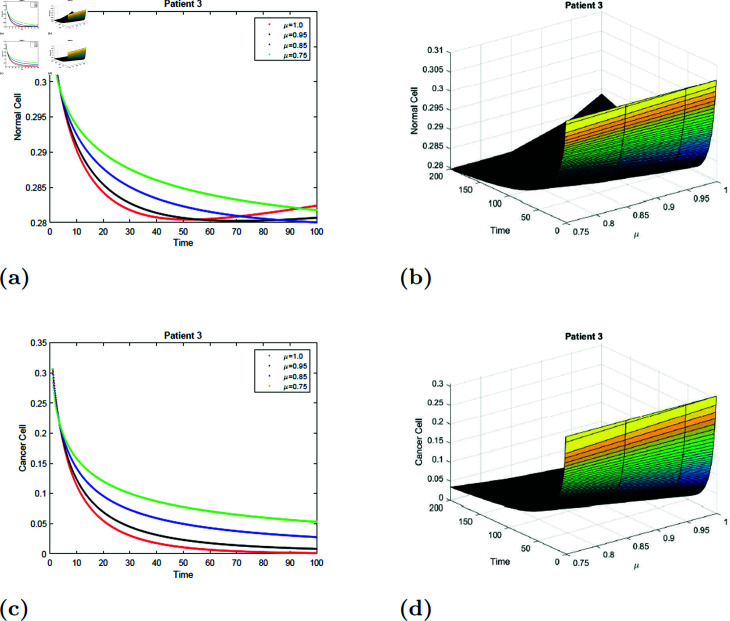
Simulation of Patient 3 at fractal fractional dimension 0.9 and γ=0.35.

The simulation of the suggested model for patient 3 with fractional orders of μ=0.75, μ=0.85, μ=0.95, and μ=1 with a fractal dimension of ν=0.9 is shown in Figs 21 and 22. Observe from Figs 21(a) and 22(a) that after 20 hours, the normal cell increases for all values of μ and converges to the equilibrium point *E*_0_. Initially, the normal cell decreases for all values of μ. Cancer cell decrease with time was shown in Figs 21(c) and 22(c) for all values of μ. Figs 21(b), 21(d), 22(b), and 22(d) depict the surface simulation of patient 3’s normal and cancerous cells. Figs 21–22 show that an increase in radiation dosage will accelerate the extinction of cancer cells while also lowering the concentration of healthy cells. The fractal-fractional model has a stronger memory impact than the fractional-order and classical models, as seen in Figs 11–22 and Tables 2–7.

## 8 Conclusion

In this study, we presented the Atangan-Baleanu fractal-fractional derivative model of radiation for the treatment of cancer. Like all the previous ones, the fractal fractional derivative of Atangana-Baleanu has several advantages. Still, its primary advantage is that it has a nonsingular and non-local kernel, which is mathematically represented by the Mittag-Leffler function sense. This function is the analytical continuation of functions defined by a series of powers. However, it has been demonstrated that said operator can better represent the complex behavior of various real-world phenomena. Both qualitatively and quantitatively, we have examined the model using the innovative operator. While the quantitative section comprises numerical results and simulations, the qualitative part also covers the existence and stability theory. Through the use of linear growth theory, we have established the existence and uniqueness of the results. The local and global stability of the fractal fractional model has been examined. The first and second derivatives of the Lyapunov function have been used to investigate the global stability of the fractal fractional model. We simulated the results for several fractional-order and fractal-dimension values using Matlab. In the mathematical modeling of the cancer model, we have found that the innovative operator yields outstanding results. Simulation results based on multiple initial values and fractional orders and fractal dimensions have been collected in cancer patient treatment. The dynamical behaviors of both cancer cells and immune cells change when we modify the fractal order. The graphical results demonstrate that the fractal-fractional model provides additional disease dynamic insights, which makes it effective as a modeling framework. The research shows that the fractal-fractional system successfully represents radiotherapy treatment of cancer while proving that changing the fractional orders and fractal dimensions creates substantial impacts on cell reaction patterns. The model presents potential for developing individualized treatment plans through improved research on how frantic structures influence non-local and memory-based interactions in cancer treatments. Scientists should conduct additional investigations about how the fractal-fractional model can model various types of cancer and different treatment protocols while using machine learning to optimize therapy parameters. Adding improved components to model the advanced relationships between cancer cells and immune systems would enable better understanding of individualized cancer treatments.
